# Continuous uniformly finite time exact disturbance observer based control for fixed-time stabilization of nonlinear systems with mismatched disturbances

**DOI:** 10.1371/journal.pone.0175645

**Published:** 2017-04-13

**Authors:** Junkang Ni, Chongxin Liu, Hang Liu

**Affiliations:** State Key Laboratory of Electrical Insulation and Power Equipment, School of Electrical Engineering, Xi’an Jiaotong University, Xi’an 710049, China; Lanzhou University of Technology, CHINA

## Abstract

This paper presents a continuous composite control scheme to achieve fixed-time stabilization for nonlinear systems with mismatched disturbances. The composite controller is constructed in two steps: First, uniformly finite time exact disturbance observers are proposed to estimate and compensate the disturbances. Then, based on adding a power integrator technique and fixed-time stability theory, continuous fixed-time stable state feedback controller and Lyapunov functions are constructed to achieve global fixed-time system stabilization. The proposed control method extends the existing fixed-time stable control results to high order nonlinear systems with mismatched disturbances and achieves global fixed-time system stabilization. Besides, the proposed control scheme improves the disturbance rejection performance and achieves performance recovery of nominal system. Simulation results are provided to show the effectiveness, the superiority and the applicability of the proposed control scheme.

## Introduction

Disturbances widely exist in many practical systems [1] and could degrade system control performance, cause damages to equipment and result in system instability. Therefore, in recent years, developing effective disturbance rejection method has become a hot topic and many control schemes have been developed, for example, see [2–4] and references therein. However, most control methods can only counteract matched disturbances. In fact, mismatched disturbances are more general disturbance form and have been found in many practical systems, for example, see [1–2, 5] and references therein. Since mismatched disturbances enter into the system through different channels from the control input, it is very (quite) difficult to design controller to eliminate their effects. Therefore, great efforts have been taken in designing control strategies to counteract the mismatched disturbances and several control schemes have been proposed, such as, integral sliding mode control [6], adaptive radial basis function (RBF) neural network based control [7], Riccati approach [8], fuzzy control [9]. However, the control methods mentioned above cannot recover the performance of nominal system. In addition, all these control methods can only achieve asymptotical stability.

Disturbance observer based control (DOBC) provides an effective approach to remove the effect of mismatched disturbances. Compared with other disturbance rejection strategies, the prominent advantage of DOBC approach lies in that it can recover the performance of nominal system. Due to this attractive feature, many DOBC methods have been proposed, for example, see [10–12]. However, these DOBC methods suffer from two main problems. One problem is that they make some restrictive assumptions that the disturbance is bounded and has constant steady state value or the disturbance is required to be bounded *H*_2_ norm, which cannot be satisfied in most engineering systems [13]. The other problem is that the presented observers are all asymptotically convergent, which implies that mismatched disturbance cannot be estimated within finite time. Actually, the longer the estimation transient is, the longer it will take the system state to converge and if the estimation period is too long, the system may escape to infinity before the observer converges. In order to overcome the drawback of these disturbance observers, a finite time disturbance observer using high order sliding mode differentiator was proposed in [14] to estimate disturbance, which shows superior performance, including stronger insensitivity to external disturbances, better disturbance rejection performance, higher convergence accuracy and finite-time convergence performance. Due to its attractive features, finite time convergent disturbance observer has been applied to compensate disturbances in many practical systems [15–16].

Finite time control method is another effective strategy to improve disturbance rejection performance. Finite time stable systems have a faster convergence rate and better robustness than asymptotical stable systems. Due to these advantages, finite time control method has received increasing attention in recent years and many meaningful results have been reported for finite time control design. The proposed control schemes are mainly based on homogeneous system technique [17–18], adding a power integrator technique [19–20] and sliding mode control design [21–22]. However, the results based on homogeneous system approach can only achieve locally finite time system stabilization and the results based on sliding mode have chattering problem due to discontinuous switching control. The feedback control scheme based on adding a power integrator technique [23] provides one feasible solution for these problems. This strategy constructs a homogeneous-based continuous differentiable Lyapunov function and employs the idea of adding a power integrator technique [24] to obtain a continuous state feedback controller such that global finite time stabilization for high order closed-loop systems can be achieved. Different from backstepping method, the feedback control scheme based on adding a power integrator technique uses feedback to dominate the nonlinear terms of the system rather than to cancel them, which can reduce the amplitude of control input [20]. Many application examples of the feedback strategy based on adding a power integrator technique have been reported, see [25–26] for example.

One drawback of finite time disturbance observer and finite time control method is that the convergence time depends on initial condition, that is, the convergence time grows unboundedly with the increase of initial condition. However, many industrial applications require severe settling time constraint. If the finite time disturbance observer and finite time control method are applied into these fields, it is necessary to assume known bounds for system state errors and adopt high gain observer and controller to guarantee convergence within prescribed time. However, this assumption does not always hold true. For example, the system states may greatly deviate from their normal value under some large disturbances. In this case, it may be hard to obtain the bound for deviation value. Besides, high gain observer amplifies measurement noise and unmodeled dynamics and high gain controller increases control costs and may result in actuator saturation. Therefore, it is important to develop a control scheme that can achieve exact disturbance estimation and system stabilization within finite time independent of initial condition.

Fixed-time stability [27] is an extension of finite time stability. In comparison with finite time stability, fixed-time stability means that system stabilization can be achieved within a limited time upper bounded by a constant independent of initial condition and the upper bound of convergence time depends only on design parameters. Due to this attractive feature, fixed-time stability has been applied to design uniform exact differentiator [28–29], design power system stable controller [30–31] and address network consensus problem [32–34]. However, there are no results about uniformly finite time exact disturbance observer and there are few results about fixed-time stable control for nonlinear system. In [35], a fixed-time nonsingular terminal sliding mode control methodology was presented for a class of second-order systems subjected to matched disturbances. However, the control method presented in [35] can only tackle matched disturbances. Moreover, it is hard to extend the presented control scheme to address high order nonlinear systems fixed-time stabilization problem. To the best of our knowledge, there are no results about control schemes to achieve fixed-time stabilization for high order nonlinear systems with mismatched disturbances.

Motivated by aforementioned discussion, a composite control method is presented to achieve fixed-time stabilization for a class of high order nonlinear systems with matched and mismatched disturbances in this paper. The composite control scheme is composed of uniformly finite time exact disturbance observer and continuous fixed-time state feedback controller, which can ensure exact disturbance estimation and system convergence within finite time bounded by a constant independent of initial condition. In comparison with the existing results of fixed-time stable control, disturbance compensation methods and finite time control, the proposed control scheme has the following features: (1) The proposed control method extends the existing fixed-time stable control methods to high order nonlinear systems with mismatched disturbances and achieves global fixed-time system stabilization. (2) The proposed control strategy overcomes the drawback of the existing finite time disturbance observers and finite time control methods, and achieves exact disturbance estimation and system stabilization within finite time upper bounded by a constant independent of initial condition. (3) The proposed control scheme removes restrictive assumption about the disturbances and can compensate a wider variety of disturbances. Besides, the proposed control scheme achieves uniformly finite time exact disturbance estimation, fixed-time system stabilization and performance recovery of nominal system. Therefore, the proposed control scheme improves the disturbance rejection performance.

The rest of this paper is organized as follows. Section 2 formulates the control problem and presents some definitions and lemmas. Main results of this paper are presented in Section 3 and simulation results verifying the effectiveness of the proposed controller are given in Section 4. Finally, the conclusion is drawn in Section 5.

## Problem formulation and preliminaries

### Problem formulation

Consider the following *n*th order single input and single output disturbed nonlinear system:

$$\begin{array}{c} {\dot{y}}_{i}=y_{i+1}+d_{i},\;\;{\dot{y}}_{n}=f\left(y\right)+g\left(y\right)u+d_{n},\;\;y_{o}=y_{1}. \end{array}$$ (1)

where *y*_*i*_, *y*_*o*_, *u*(*i* = 1, 2, …, *n*) are state variables, system output and control input; *f*(*y*) and *g*(*y*) are known smooth nonlinear functions. The system has disturbances in all channels with *d*_*i*_(*i* = 1, 2, …, *n* − 1) being mismatched disturbances and *d*_*n*_ being matched one and the disturbances are unknown and unmeasurable. The disturbances are supposed to satisfy the following assumption:

**Assumption 1** The disturbance *d*_*i*_(*t*) in System (1) is *n*–*th* order differentiable and $d_{i}^{\left(n\right)}$ has a positive Lipschitz constant *L*_*i*_, i.e., $|d_{i}^{\left(n\right)}|<L_{i}$.

**Remark 1** Matched disturbances are disturbances that enter into the system through the same channel as the control input, while mismatched disturbances are disturbances that enter into the system through different channels from the control input. For example, in the last channel of System (1), i.e., *y*_*n*_, since disturbance *d*_*n*_ and control input *u* appear simultaneously, the disturbance *d*_*n*_ is called matched disturbance. In other channels of System (1), the disturbances *d*_*i*_(*i* = 1, 2, …, *n* − 1) appear but no control input appears, therefore, the disturbances *d*_*i*_(*i* = 1, 2, …, *n* − 1) are called mismatched disturbances.

**Remark 2** System (1) is a Brunovsky system with matched and mismatched disturbances. Many practical systems have the same form as System (1) or can be transformed into System (1), such as, flexible joint manipulator [36], maglev suspension system [37], DC-DC buck power converter [5], permanent magnet synchronous motor [26], power system [1].

**Remark 3** The disturbances considered in this paper are more general than many DOBC methods, such as [10–12]. In fact, a wide variety of disturbances, such as constant disturbance, ramp disturbance, sinusoidal and polynomial disturbance, satisfy Assumption 1.

The initial condition of System (1) is assumed to be unknown and the problem addressed in this paper is to design the control *u* such that the influence of disturbances can be rejected from the output channel and the system output *y*_*o*_ can be regulated to the origin within finite time independent of initial condition.

### Fixed-time stability

Consider the following differential equation system:

$$\begin{array}{c} \dot{x}\left(t\right)=f\left(x\left(t\right)\right),\;\;x\left(0\right)=x_{0}. \end{array}$$ (2)

where *x* ∈ *R* and *f*: *R*_+_ × *R*^*n*^ → *R*^*n*^ is a nonlinear function. Suppose that the origin is an equilibrium point of Eq (2).

**Definition 1** [38], [39]: The origin of System (2) is a finite time stable equilibrium if the origin is Lyapunov stable and there exists a function *T*: *R*^*n*^ → *R*^+^, called the settling time function, such that for every *x*_0_ ∈ *R*^*n*^, the solution *x*(*t*, *x*_0_) of System (2) is defined on [0, *T*(*x*_0_)), *x*(*t*, *x*_0_) ∈ *R*^*n*^, for all *t* ∈ [0, *T*(*x*_0_)), and $\underset{t\to T(x_{0})}{\text{lim}}x(t,x_{0})=0$.

**Definition 2** [27]: The origin of System (2) is said to be a fixed-time stable equilibrium point if it is globally finite-time stable with bounded convergence time *T*(*x*_0_), that is, there exists a bounded positive constant *T*_max_ such that *T*(*x*_0_) < *T*_max_ satisfies.

**Lemma 1** [27] Suppose there exists a positive definite *C*^1^ function *V*(*x*) : *U* → *R*, positive real numbers *α* and *β*, positive odd integers *m*, *n*, *p*, *q* that satisfy *m* > *n*, *p* < *q* and an open neighborhood *U*_0_ ⊂ *U* of the origin, such that $\dot{V}+\alpha V^{m/n}+\beta V^{p/q}\leq 0$, *x* ∈ *U*_0_∖{0}. Then the origin of System (2) is fixed-time stable and the convergence time is bounded by $T<T_{\max}=\frac{1}{\alpha }\frac{n}{m-n}+\frac{1}{\beta }\frac{q}{q-p}$. If *U* = *U*_0_ = *R*^*n*^, the origin is a globally fixed-time stable equilibrium of System (2).

**Remark 4** The upper bound of convergence time relies only on the design parameters *α*, *β*, *m*, *n*, *p*, *q*, which implies even if the initial condition is unavailable in advance or becomes infinity, the system can be stabilized within a bounded time and the convergence time can be assigned in advance.

### Homogeneity property

**Definition 3** [40] Let *r* = (*r*_1_, …, *r*_*n*_) be a generalized weight vector with *r*_*i*_ > 0. The dilation associated to the weight vector *r* is: $\Lambda_{r}:\left(x_{1},x_{2},...,x_{n}\right)\mapsto \left(\lambda^{r_{1}}x_{1},...,\lambda^{r_{n}}x_{n}\right)$ for *λ* > 0. A vector field *f* is said to be a homogeneous function of degree *m* with respect to a generalized weight *r* iff for all *x* ∈ *R*^*n*^ and *λ* > 0, we have $\lambda^{-m}\Lambda_{r}^{-1}f\left(\Lambda_{r}x\right)=f\left(x\right)$.

Homogeneity property can be used to obtain finite time stability property and uniform convergence property.

Finite time convergence means that exact convergence can be achieved within finite time. The notion of homogeneity can be used to obtain finite time stability property as follows:

**Lemma 2** [39] If *f*: *R*^*n*^ → *R*^*n*^ is a homogeneous vector field of degree *k* < 0 and locally attractive, then *f* is globally finite-time stable (FTS).

Uniform convergence property means that for any initial condition, the convergence time is uniformly bounded by a constant. Based on homogeneity property, the definition of uniform convergence is given as follows:

**Definition 4** [29] Consider the following system:

$$\begin{array}{c} \dot{\tilde{x}}=f\left(\tilde{x}\right)+g\left(\tilde{x},w\right),\;\;\;\tilde{x}\left(0\right)={\tilde{x}}_{0} \end{array}$$ (3)

where *w* is external disturbance uniformly bounded by a constant, $g(\tilde{x},w)$ can be considered as a disturbance term to the nominal part $f(\tilde{x})$. System (3) is said to be practically uniformly convergent w.r.t. initial value if there exist positive constants *T* and *r* such that for all ${\tilde{x}}_{0}\in R^{n}$, $∥\tilde{x}\left(t\right)∥\leq r$ holds for all *t* ≥ *T*.

**Lemma 3** [29] System (3) is practically uniformly convergent w.r.t. initial value ${\tilde{x}}_{0}$ if (i) its origin is globally asymptotically stable when *g* ≡ 0; (ii) *f* is a continuous homogeneous vector field of degree *m* > 0; (iii) disturbance *w* is uniformly bounded.

Combine finite time stability property and uniform convergence property, and the concept of uniformly finite time exact can be given as follows:

**Lemma 4** [29] System (3) is said to be uniformly finite time exact, if disturbance *w* is uniformly bounded and there exists a constant *T* independent of initial condition ${\tilde{x}}_{0}\in R^{n}$ such that for any initial condition $\tilde{x}\left(0\right)$, system trajectory converges to the origin after *T*.

### Mathematical lemmas

**Lemma 5** [41]: For a ratio of positive odd integers *p* ∈ (0, 1) and real variables *x*, *y*, the following inequality holds:

$$\begin{array}{c} |x^{p}-y^{p}|\leq 2^{1-p}{\left|x-y\right|}^{p} \end{array}$$ (4)

**Lemma 6** [42]: For positive real numbers *c*, *d* and real variables *x*, *y*, the following inequality holds:

$$\begin{array}{c} {\left|x\right|}^{c}{\left|y\right|}^{d}\leq \frac{c}{c+d}{\left|x\right|}^{c+d}+\frac{d}{c+d}{\left|y\right|}^{c+d} \end{array}$$ (5)

**Lemma 7** [43]: For any positive real numbers *b*, *m*, *n* and continuous functions *x*, *y*, *z* ≥ 0, one has:

$$\begin{array}{c} {\left|x\right|}^{n}{\left|y\right|}^{m}z\leq b{\left|x\right|}^{m+n}+\frac{m}{n+m}{\left(\frac{n}{b(n+m)}\right)}^{n/m}{\left|y\right|}^{n+m}z^{(n+m)/m} \end{array}$$ (6)

**Lemma 8** [44]: For any nonnegative real numbers *ξ*_1_, *ξ*_2_, …, *ξ*_*n*_ and 0 < *p* ≤ 1, the following inequality holds:

$$\begin{array}{c} \sum_{i=1}^{n}\xi_{i}^{p}\geq {\left(\sum_{i=1}^{n}\xi_{i}\right)}^{p} \end{array}$$ (7)

**Lemma 9** [31]: For any nonnegative real numbers *ξ*_1_, *ξ*_2_, …, *ξ*_*n*_ and *p* > 1, the following inequality holds:

$$\begin{array}{c} \sum_{i=1}^{n}\xi_{i}^{p}\geq n^{1-p}{\left(\sum_{i=1}^{n}\xi_{i}\right)}^{p} \end{array}$$ (8)

**Lemma 10** If 0 ≤ *τ* ≤ 1, for any real variable *ξ*_1_, the following inequality holds:

$$\begin{array}{c} {\left|\xi_{1}\right|}^{2-2\tau }\leq 1+\xi_{1}^{2} \end{array}$$ (9)

*Proof*: Since 0 ≤ *τ* ≤ 1, we have 0 ≤ 2 − 2*τ* ≤ 2. If |*ξ*_1_| ≤ 1, we have |*ξ*_1_|^2−2*τ*^ ≤ 1 and if |*ξ*_1_| > 1, one has ${\left|\xi_{1}\right|}^{2-2\tau }\leq \xi_{1}^{2}$. Thus, for any real variable *ξ*_1_, we have ${\left|\xi_{1}\right|}^{2-2\tau }\leq 1+\xi_{1}^{2}$.

## Main results

Since the disturbances are unknown and unmeasurable, disturbance observers are first constructed to estimate the disturbances. Using the idea of uniform finite time HOSM differentiator in [29] and finite time disturbance observer in [14], we propose the following uniformly finite time exact disturbance observer:

$$\begin{array}{ccc} {\dot{z}}_{0i} & = & v_{0i}+h_{i},{\dot{z}}_{ji}=v_{ji},\ldots ,{\dot{z}}_{ni}=v_{ni}\\ v_{0i} & = & -k_{0i}\theta {\left|z_{0i}-y_{i}\right|}^{n/(n+1)}\text{sign}\left(z_{0i}-y_{i}\right)\\ & & -k_{0i}\left(1-\theta \right){\left|z_{0i}-y_{i}\right|}^{\left(n+1+\alpha )/(n+1\right)}\text{sign}\left(z_{0i}-y_{i}\right)\\ & & +z_{1i}\\ v_{ji} & = & -k_{ji}\theta {\left|z_{ji}-v_{(j-1)i}\right|}^{\left(n-j)/(n-j+1\right)}\text{sign}\left(z_{ji}-v_{(j-1)i}\right)\\ & & -k_{ji}\left(1-\theta \right){\left|z_{0i}-y_{i}\right|}^{\left(n+1+(j+1)\alpha )/(n+1\right)}\text{sign}\left(z_{0i}-y_{i}\right)+z_{(j+1)i}\\ v_{ni} & = & -k_{ni}\theta \text{sign}\left(z_{ni}-v_{(n-1)i}\right)-k_{ni}\left(1-\theta \right){\left|z_{0i}-y_{i}\right|}^{1+\alpha }\text{sign}\left(z_{0i}-y_{i}\right) \end{array}$$ (10)

where *i* = 1, …, *n*, *j* = 1, …, *n* − 1, *h*_*i*_ = *y*_*i*+1_ for *i* = 1, …, *n* − 1, *h*_*n*_ = *f*(*y*) + *g*(*y*)*u*, *k*_0*i*_, …, *k*_*ni*_ and *α* are observer coefficients to be designed, *θ* is a function to be designed and *z*_0*i*_, *z*_1*i*_, …, *z*_*ni*_ are estimation for *y*_*i*_, $d_{i},\ldots ,d_{i}^{\left(n-1\right)}$ respectively.

**Theorem 1** The disturbance observer Eq (10) is uniformly finite time exact, i.e., exact disturbance estimation can be achieved within finite time *t*_1_ upper bounded by a constant *T*_1_ independent of initial estimation error, if its parameters satisfy the following conditions:

1) *α* is a sufficiently small positive constant;

2) The observer coefficients *k*_*ji*_(*j* = 0, …, *n*) are assigned such that the following matrix is Hurwitz:

$$\begin{array}{c} A=[\begin{array}{ccccc} -k_{0i} & 1 & 0 & \ldots & 0\\ -k_{1i} & 0 & 1 & \ldots & 0\\ \ldots & \ldots & \ldots & \ldots & \ldots \\ -k_{ni} & 0 & 0 & \ldots & 0 \end{array}] \end{array}$$ (11)

3) The observer coefficients *k*_*ji*_(*j* = 0, …, *n*) are selected according to the condition $|d_{i}^{\left(n\right)}|<L_{i}$.

4) The function *θ*: [0, ∞) → {0, 1} is selected as:

$$\begin{array}{c} \theta \left(t\right)=\{\begin{array}{cc} 0 & if\;t\leq T_{u}\\ 1 & if\;t>T_{u} \end{array} \end{array}$$

where *T*_*u*_ is switching time, a design parameter that is typically selected through numerical simulations and trial and error.

*Proof*: See Appendix A.

**Remark 5** Theorem 1 shows that the proposed disturbance observer can achieve exact disturbance estimation within uniformly bounded time *T*_1_ independent of initial estimation error and the bound of estimation time can be obtained through numerical simulation.

Substituting the estimated disturbance value into the dynamics of System (1), one has

$$\begin{array}{c} {\dot{y}}_{i}=y_{i+1}+z_{1i}-\sigma_{1i},{\dot{y}}_{n}=f\left(y\right)+g\left(y\right)u+z_{1n}-\sigma_{1n} \end{array}$$ (12)

For *t* ≥ *t*_1_, the disturbance estimation errors *σ*_1*i*_ converge to zero and System (12) reduces to:

$$\begin{array}{c} {\dot{y}}_{i}=y_{i+1}+z_{1i},{\dot{y}}_{n}=f\left(y\right)+g\left(y\right)u+z_{1n} \end{array}$$ (13)

### Continuous fixed-time state feedback control design

To design continuous fixed-time state feedback controller, we introduce coordinate transformation ${\bar{y}}_{k}={\dot{\bar{y}}}_{k-1}=y_{k}+\sum_{j=1}^{k-1}z_{(k-j)j},\left(k=2,3,\ldots ,n\right)$. Under this coordinate transformation, the System (13) becomes:

$$\begin{array}{c} {\dot{y}}_{1}={\bar{y}}_{2},{\dot{\bar{y}}}_{i}={\bar{y}}_{i+1},\ldots ,{\dot{\bar{y}}}_{n}=f\left(y\right)+g\left(y\right)u+\sum_{j=1}^{n}z_{(n+1-j)j} \end{array}$$ (14)

Now, the System (14) is transformed into a Brunovsky system. A composite controller using the method of adding a power integrator will be designed for Brunovsky System (14) and fixed-time stability analysis of proposed control scheme will be given. To construct this controller, we first define:*q*_1_ = 1, $1+\frac{1}{q_{i+1}}=\tau +\frac{1}{q_{i}}$, 0 < *τ* < 1, *q*_*j*_ > 1(*j* = 2, 3, …, *n*) and ${\left[\cdot \right]}^{\alpha }={\left|\cdot \right|}^{\alpha }\text{sign}\left(\cdot \right),\alpha >0$.

Step 1: Choose the following *C*^1^ Lyapunov function $V_{1}=\frac{1}{2}y_{1}^{2}$ and the derivative of *V*_1_ along the trajectory of System (14) is:

$$\begin{array}{c} {\dot{V}}_{1}=y_{1}{\dot{y}}_{1}=y_{1}{\bar{y}}_{2}^{*}+y_{1}\left({\bar{y}}_{2}-{\bar{y}}_{2}^{*}\right) \end{array}$$ (15)

where ${\bar{y}}_{2}^{*}$ is a virtual control law. Define *ξ*_1_ = *y*_1_ and the virtual control law can be designed as:

$$\begin{array}{c} {\bar{y}}_{2}^{*}=-\left(k_{1}+l_{1}\left(1+\xi_{1}^{2}\right)\right){\left[\xi_{1}\right]}^{1/q_{2}}=-\gamma_{1}\left(\xi \right){\left[\xi_{1}\right]}^{1/q_{2}} \end{array}$$ (16)

where *k*_1_ > 0, *l*_1_ > 0, and $\gamma_{1}\left(\xi \right)=k_{1}+l_{1}\left(1+\xi_{1}^{2}\right)$

By Lemma 5 and Lemma 6, one has

$$\begin{array}{ccc} \left|{\bar{y}}_{2}-{\bar{y}}_{2}^{*}||y_{1}\right| & = & |{\left({\bar{y}}_{2}^{q_{2}}\right)}^{1/q_{2}}-{\left({\bar{y}}_{2}^{*q_{2}}\right)}^{1/q_{2}}||\xi_{1}|\leq 2^{1-1/q_{2}}{\left|\xi_{2}\right|}^{1/q_{2}}\left|\xi_{1}\right|\\ & \leq & \frac{2^{1-1/q_{2}}}{1+\tau }{\left|\xi_{1}\right|}^{1+\tau }+\frac{2^{1-1/q_{2}}\tau }{1+\tau }{\left|\xi_{2}\right|}^{1+\tau }=c_{11}{\left|\xi_{1}\right|}^{1+\tau }+c_{12}{\left|\xi_{2}\right|}^{1+\tau } \end{array}$$ (17)

where $\xi_{2}={\bar{y}}_{2}^{q_{2}}-{\bar{y}}_{2}^{*q_{2}}$.

Substituting Eqs (16) and (17) into Eq (15) and utilizing Lemma 10, one obtains:

$$\begin{array}{c} {\dot{V}}_{1}\leq -k_{1}{\left|\xi_{1}\right|}^{1+\tau }-l_{1}{\left|\xi_{1}\right|}^{3-\tau }+c_{11}{\left|\xi_{1}\right|}^{1+\tau }+c_{12}{\left|\xi_{2}\right|}^{1+\tau } \end{array}$$ (18)

Inductive step: Suppose that at step *i*, there exists a function $\gamma_{1}\left(\xi \right)=k_{1}+l_{1}\left(1+\xi_{1}^{2}\right)$ and functions $\gamma_{j}\left(\xi \right)=k_{j}+l_{j}\left(1+\xi_{j}^{2}\right)+\chi_{j-1}\left(\xi \right),j=2,3,\ldots ,i$, such that the following holds:

$$\begin{array}{ccc} {\dot{V}}_{i} & \leq & -\sum_{j=1}^{i}k_{j}{\left|\xi_{j}\right|}^{1+\tau }-\sum_{j=1}^{i}l_{j}{\left|\xi_{j}\right|}^{3-\tau }+\sum_{j=1}^{i}c_{j1}{\left|\xi_{1}\right|}^{1+\tau }+\sum_{j=1}^{i}c_{j2}{\left|\xi_{2}\right|}^{1+\tau }\\ & & +\sum_{j=2}^{i}c_{j3}{\left|\xi_{3}\right|}^{1+\tau }+\cdots +c_{i(i+1)}{\left|\xi_{i+1}\right|}^{1+\tau } \end{array}$$ (19)

where *V*_*i*_ = *V*_*i*−1_ + *W*_*i*_ is positive definite and proper with $W_{i}=\int_{{\bar{y}}_{i}^{*}}^{{\bar{y}}_{i}}{\left[s^{q_{i}}-{\bar{y}}_{i}^{*q_{i}}\right]}^{2-1/q_{i}}ds$ and

$$\begin{array}{cc} y_{1}^{*}=0, & \;\;\;\xi_{1}=y_{1}\\ {\bar{y}}_{2}^{*}=-\gamma_{1}\left(\xi \right){\left[\xi_{1}\right]}^{1/q_{2}}, & \;\;\;\xi_{2}={\bar{y}}_{2}^{q_{2}}-{\bar{y}}_{2}^{*q_{2}}\\ \cdots , & \;\;\;\cdots \\ {\bar{y}}_{i+1}^{*}=-\gamma_{i}\left(\xi \right){\left[\xi_{i}\right]}^{1/q_{i+1}}, & \;\;\;\xi_{i+1}={\bar{y}}_{i+1}^{q_{i+1}}-{\bar{y}}_{i+1}^{*q_{i+1}} \end{array}$$ (20)

In what follows, we will show that Eq (19) also holds at step *i* + 1. To this end, the following Lyapunov function is considered:

$$\begin{array}{c} V_{i+1}=V_{i}+W_{i+1} \end{array}$$ (21)

where $W_{i+1}=\int_{{\bar{y}}_{i+1}^{*}}^{{\bar{y}}_{i+1}}{\left[s^{q_{i+1}}-{\bar{y}}_{i+1}^{*q_{i+1}}\right]}^{2-1/q_{i+1}}ds$

The time derivative of Lyapunov function Eq (21) is:

$$\begin{array}{c} {\dot{V}}_{i+1}={\dot{V}}_{i}+{\dot{W}}_{i+1} \end{array}$$ (22)

where

$$\begin{array}{ccc} {\dot{W}}_{i+1} & = & {\bar{y}}_{i+2}{\left[\xi_{i+1}\right]}^{2-1/q_{i+1}}+\sum_{j=1}^{i}\frac{\partial W_{i+1}}{\partial {\bar{y}}_{j}}{\dot{\bar{y}}}_{j}\\ & = & {\bar{y}}_{i+2}^{*}{\left[\xi_{i+1}\right]}^{2-1/q_{i+1}}+\left({\bar{y}}_{i+2}-{\bar{y}}_{i+2}^{*}\right){\left[\xi_{i+1}\right]}^{2-1/q_{i+1}}+\sum_{j=1}^{i}\frac{\partial W_{i+1}}{\partial {\bar{y}}_{j}}{\dot{\bar{y}}}_{j}\\ & \leq & {\bar{y}}_{i+2}^{*}{\left[\xi_{i+1}\right]}^{2-1/q_{i+1}}+\left|{\bar{y}}_{i+2}-{\bar{y}}_{i+2}^{*}\right|{\left|\xi_{i+1}\right|}^{2-1/q_{i+1}}+\sum_{j=1}^{i}\frac{\partial W_{i+1}}{\partial {\bar{y}}_{j}}{\dot{\bar{y}}}_{j} \end{array}$$ (23)

Using Lemma 5 and Lemma 6, the second term in Eq (23) can be estimated as:

$$\begin{array}{ccc} & & |{\bar{y}}_{i+2}-{\bar{y}}_{i+2}^{*}|{\left|\xi_{i+1}\right|}^{2-1/q_{i+1}}\\ & & \;\leq 2^{1-1/q_{i+2}}{\left|{\bar{y}}_{i+2}^{q_{i+2}}-{\bar{y}}_{i+2}^{*q_{i+2}}\right|}^{1/q_{i+2}}{\left|\xi_{i+1}\right|}^{2-1/q_{i+1}}\\ & & \;=2^{1-1/q_{i+2}}{\left|\xi_{i+2}\right|}^{1/q_{i+2}}{\left|\xi_{i+1}\right|}^{2-1/q_{i+1}}\\ & & \;\leq \frac{2^{1-1/q_{i+2}}/q_{i+2}}{1+\tau }{\left|\xi_{i+2}\right|}^{1+\tau }+\frac{2^{1-1/q_{i+2}}\left(2-1/q_{i+1}\right)}{1+\tau }{\left|\xi_{i+1}\right|}^{1+\tau }\\ & & \;=c_{\left(i+1)(i+2\right)}{\left|\xi_{i+2}\right|}^{1+\tau }+c_{\left(i+1)(i+1\right)}{\left|\xi_{i+1}\right|}^{1+\tau } \end{array}$$ (24)

To estimate the last term in Eq (23), we introduce the following proposition, whose proof are given in Appendix B

**Proposition 1** There exists a function *χ*_*i*_(*ξ*) and functions *c*_(*i* + 1)*j*_, *j* = 1, 2, …, *i* such that

$$\begin{array}{c} \sum_{j=1}^{i}\frac{\partial W_{i+1}}{\partial {\bar{y}}_{j}}{\dot{\bar{y}}}_{j}\leq \sum_{j=1}^{i}c_{(i+1)j}{\left|\xi_{j}\right|}^{1+\tau }+\chi_{i}\left(\xi \right){\left|\xi_{i+1}\right|}^{1+\tau } \end{array}$$ (25)

The virtual control can be designed as:

$$\begin{array}{c} {\bar{y}}_{i+2}^{*}=-\left(k_{i+1}+l_{i+1}\left(1+\xi_{i+1}^{2}\right)+\chi_{i}\left(\xi \right)\right){\left[\xi_{i+1}\right]}^{1/q_{i+2}}=-\gamma_{i+1}\left(\xi \right){\left[\xi_{i+1}\right]}^{1/q_{i+2}} \end{array}$$ (26)

Substituting Eqs (24)–(26) into Eq (23), one has:

$$\begin{array}{c} {\dot{W}}_{i+1}\leq -k_{i+1}{\left|\xi_{i+1}\right|}^{1+\tau }-l_{i+1}{\left|\xi_{i+1}\right|}^{3-\tau }+\sum_{j=1}^{i+2}c_{(i+1)j}{\left|\xi_{j}\right|}^{1+\tau } \end{array}$$ (27)

Substituting Eqs (19) and (27) into Eq (22), the derivative of Lyapunov function *V*_*i*+1_ can be obtained as:

$$\begin{array}{ccc} {\dot{V}}_{i+1} & \leq & -\sum_{j=1}^{i+1}k_{j}{\left|\xi_{j}\right|}^{1+\tau }-\sum_{j=1}^{i+1}l_{j}{\left|\xi_{j}\right|}^{3-\tau }+\sum_{j=1}^{i+1}c_{j1}{\left|\xi_{1}\right|}^{1+\tau }+\sum_{j=1}^{i+1}c_{j2}{\left|\xi_{2}\right|}^{1+\tau }\\ & & +\sum_{j=2}^{i+1}c_{j3}{\left|\xi_{3}\right|}^{1+\tau }+\cdots +c_{\left(i+1)(i+2\right)}{\left|\xi_{i+2}\right|}^{1+\tau } \end{array}$$ (28)

This completes the inductive proof.

Step n: According to inductive proof, at step n, we can design the control input as:

$$\begin{array}{ccc} u & = & \frac{1}{g(y)}\left({\bar{y}}_{n+1}^{*}-\sum_{j=1}^{n}z_{(n+1-j)j}-f\left(y\right)\right)\\ & = & \frac{1}{g(y)}\left(-\left(k_{n}+l_{n}\left(1+\xi_{n}^{2}\right)+\chi_{n-1}\left(\xi \right)\right){\left[\xi_{n}\right]}^{1/q_{n+1}}-\sum_{j=1}^{n}z_{(n+1-j)j}-f\left(y\right)\right)\\ & = & \frac{1}{g(y)}\left(-\gamma_{n}\left(\xi \right){\left[\xi_{n}\right]}^{1/q_{n+1}}-\sum_{j=1}^{n}z_{(n+1-j)j}-f\left(y\right)\right) \end{array}$$ (29)

### Stability analysis

**Theorem 2** Suppose that the disturbances in System (1) satisfy Assumption 1. Then the composite control scheme consisting of uniformly finite time exact disturbance observer Eq (10) and continuous fixed-time state feedback control law Eq (29) can achieve global fixed-time stabilization for disturbed nonlinear System (1).

*Proof*: The proof process can be divided into two parts. The first part will prove the continuous fixed-time state feedback control law Eq (29) can achieve fixed-time stabilization for System (1) when *t* > *t*_1_ and the second part will show the states of the System (12) and the observer Eq (10) keep bounded at any time interval [0, *t*_1_].

For the first part proof, the Lyapunov function can be constructed as

$$\begin{array}{c} V_{n}=V_{1}+\sum_{i=2}^{n}W_{i}=\frac{1}{2}y_{1}^{2}+\sum_{i=2}^{n}\int_{{\bar{y}}_{i}^{*}}^{{\bar{y}}_{i}}{\left[s^{q_{i}}-{\bar{y}}_{i}^{*q_{i}}\right]}^{2-1/q_{i}}ds \end{array}$$ (30)

**Remark 6** Similar to [42] and [45], it can be proved that the considered Lyapunov function *V*_*n*_ is positive definite.

Following the same line of inductive proof, it is straightforward to see that Eq (19) holds for *i* = *n* with a series of virtual controllers defined in Eq (20). Since ${\bar{y}}_{n+1}^{*}={\bar{y}}_{n+1}$, we have *ξ*_*n*+1_ = 0 and the time derivative of Lyapunov function *V*_*n*_ can be given as:

$$\begin{array}{ccc} {\dot{V}}_{n} & \leq & -\sum_{j=1}^{n}k_{j}{\left|\xi_{j}\right|}^{1+\tau }-\sum_{j=1}^{n}l_{j}{\left|\xi_{j}\right|}^{3-\tau }+\sum_{j=1}^{n}c_{j1}{\left|\xi_{1}\right|}^{1+\tau }+\sum_{j=1}^{n}c_{j2}{\left|\xi_{2}\right|}^{1+\tau }\\ & & +\sum_{j=2}^{n}c_{j3}{\left|\xi_{3}\right|}^{1+\tau }+\cdots +\sum_{j=n-1}^{n}c_{jn}{\left|\xi_{n}\right|}^{1+\tau }\\ & = & -\left(k_{1}-\sum_{j=1}^{n}c_{j1}\right){\left|\xi_{1}\right|}^{1+\tau }-\left(k_{2}-\sum_{j=1}^{n}c_{j2}\right){\left|\xi_{2}\right|}^{1+\tau }\\ & & -\sum_{j=3}^{n}\left(k_{j}-\sum_{i=j-1}^{n}c_{ij}\right){\left|\xi_{j}\right|}^{1+\tau }-\sum_{j=1}^{n}l_{j}{\left|\xi_{j}\right|}^{3-\tau } \end{array}$$ (31)

If the parameters *k*_*i*_, *l*_*i*_(*i* = 1, ⋯, *n*) can be selected such that $k_{1}>\sum_{j=1}^{n}c_{j1}$, $k_{2}>\sum_{j=1}^{n}c_{j2}$, $k_{j}>\sum_{i=j-1}^{n}c_{ij}\left(j=3,\cdots ,n\right),l_{i}>0\left(i=1,\cdots ,n\right)$ hold, the derivative of Lyapunov function *V*_*n*_ is negative definite and the System (14) can be stabilized asymptotically. Specifically, the derivative of Lyapunov function *V*_*n*_ can also be expressed as:

$$\begin{array}{c} {\dot{V}}_{n}\leq -C\sum_{j=1}^{n}{\left|\xi_{j}\right|}^{1+\tau }-L\sum_{j=1}^{n}{\left|\xi_{j}\right|}^{3-\tau } \end{array}$$ (32)

where $C=\min\left\{k_{1}-\sum_{j=1}^{n}c_{j1},k_{2}-\sum_{j=1}^{n}c_{j2},k_{j}-\sum_{i=j-1}^{n}c_{ij}\right\}\left(j=3,4,\cdots ,n\right)$, *L* = min{*l*_*i*_}(*i* = 1, 2, ⋯, *n*). Using mean value theorem for integral and Lemma 5, it can be verified that:

$$\begin{array}{ccc} V_{n} & = & \frac{1}{2}y_{1}^{2}+\sum_{i=2}^{n}\int_{{\bar{y}}_{i}^{*}}^{{\bar{y}}_{i}}{\left[s^{q_{i}}-{\bar{y}}_{i}^{*q_{i}}\right]}^{2-1/q_{i}}ds\leq \frac{1}{2}y_{1}^{2}+\sum_{i=2}^{n}{\left|\xi_{i}\right|}^{2-1/q_{i}}\left|{\bar{y}}_{i}-{\bar{y}}_{i}^{*}\right|\\ & \leq & \frac{1}{2}{\left|\xi_{1}\right|}^{2}+\sum_{i=2}^{n}2^{1-1/q_{i}}{\left|\xi_{i}\right|}^{2}\leq D\sum_{i=1}^{n}{\left|\xi_{i}\right|}^{2} \end{array}$$ (33)

where $D=\max\left\{\frac{1}{2},2^{1-1/q_{i}}\right\}\left(i=2,3,\ldots ,n\right)$. Since 0 < *τ* < 1, we have (1 + *τ*)/2 < 1 and (3 − *τ*)/2 > 1. According to Lemma 8 and Lemma 9, we can derive

$$\begin{array}{c} {\dot{V}}_{n}+\frac{C}{D^{(1+\tau )/2}}V_{n}^{(1+\tau )/2}+Ln^{1-(3-\tau )/2}{\left(\frac{V_{n}}{D}\right)}^{(3-\tau )/2}\leq {\dot{V}}_{n}+C\sum_{j=1}^{n}{\left|\xi_{j}\right|}^{1+\tau }+L\sum_{j=1}^{n}{\left|\xi_{j}\right|}^{3-\tau }\leq 0 \end{array}$$ (34)

If the parameter *τ* is selected as *τ* = (2*k* − 3)/(2*k* + 1), the numerator and denominator of the fractional power (1 + *τ*)/2 and (3 − *τ*)/2 will be both odd. According to Lemma 1, the System (14) can be stabilized within finite time and the upper bound of convergence time can be estimated as:

$$\begin{array}{c} T<T_{\max}=\frac{D^{\left(2k+3)/(2k+1\right)}}{Ln^{1-(2k+3)/(2k+1)}}\frac{2k+1}{2}+\frac{D^{\left(2k-1)/(2k+1\right)}}{C}\frac{2k+1}{2} \end{array}$$ (35)

This follows that the proposed control scheme can achieve global fixed-time system stabilization. Next, we will show the states of the System (12) and the observer Eq (10) keep bounded at any time interval [0, *t*_1_]. The considered Lyapunov function is:

$$\begin{array}{c} M=\frac{1}{2}\left(y_{1}^{2}+y_{2}^{2}+\cdots +y_{n}^{2}+z_{0}^{T}z_{0}+z_{1}^{T}z_{1}+\cdots +z_{n}^{T}z_{n}\right) \end{array}$$ (36)

Let us first consider *t* ∈ [*T*_*u*_, *t*_1_]. In this case, *θ* in Eq (10) equals to one. The time derivative of Lyapunov function *M* along Eqs (12) and (10) can be given as:

$$\begin{array}{ccc} \dot{M} & = & y_{1}\left(y_{2}+z_{11}-\sigma_{11}\right)+y_{2}\left(y_{3}+z_{12}-\sigma_{12}\right)+\cdots +y_{n}\left(f\left(y\right)+g\left(y\right)u+z_{1n}-\sigma_{1n}\right)\\ & & +\sum_{i=1}^{n-1}z_{0i}\left(-k_{0i}{\left|z_{0i}-y_{i}\right|}^{n/(n+1)}\text{sign}\left(z_{0i}-y_{i}\right)+z_{1i}+y_{i+1}\right)\\ & & +z_{0n}\left(-k_{0n}{\left|z_{0n}-y_{n}\right|}^{n/(n+1)}\text{sign}\left(z_{0n}-y_{n}\right)+z_{1n}+f\left(y\right)+g\left(y\right)u\right)\\ & & +\sum_{i=1}^{n}z_{1i}\left(-k_{1i}{\left|z_{1i}-v_{0i}\right|}^{(n-1)/n}\text{sign}\left(z_{1i}-v_{0i}\right)+z_{2i}\right)+\cdots \\ & & +\sum_{i=1}^{n}z_{ni}\left(-k_{ni}\text{sign}\left(z_{ni}-v_{(n-1)i}\right)\right) \end{array}$$ (37)

Note that

$$\begin{array}{ccc} f(y)+g(y)u & = & -\gamma_{n}\left(\xi \right){\left[{\bar{y}}_{n}^{q_{n}}-{\bar{y}}_{n}^{*q_{n}}\right]}^{1/q_{n+1}}-\sum_{j=1}^{n}z_{(n+1-j)j}\\ & \leq & \gamma_{n}\left(\xi \right)\left({\left|{\bar{y}}_{n}\right|}^{\frac{q_{n}}{q_{n+1}}}+{\left|{\bar{y}}_{n}^{*}\right|}^{\frac{q_{n}}{q_{n+1}}}\right)+\sum_{j=1}^{n}\left|z_{(n+1-j)j}\right|\\ & \leq & \gamma_{n}\left(\xi \right)({\left|y_{n}\right|}^{\frac{q_{n}}{q_{n+1}}}+\sum_{j=1}^{n-1}{\left|z_{(n-j)j}\right|}^{\frac{q_{n}}{q_{n+1}}}+\cdots \\ & & +\gamma_{n-1}^{\frac{q_{n}}{q_{n+1}}}\gamma_{n-2}^{\frac{q_{n-1}}{q_{n+1}}}\cdots \gamma_{1}^{\frac{q_{2}}{q_{n+1}}}{\left|y_{1}\right|}^{\frac{1}{q_{n+1}}})+\sum_{j=1}^{n}\left|z_{(n+1-j)j}\right| \end{array}$$ (38)

$$\begin{array}{c} z_{0i}\left(-k_{0i}{\left|z_{0i}-y_{i}\right|}^{n/(n+1)}\text{sign}\left(z_{0i}-y_{i}\right)\right)\leq k_{0i}\left|z_{0i}\right|\left({\left|z_{0i}\right|}^{n/(n+1)}+{\left|y_{i}\right|}^{n/(n+1)}\right) \end{array}$$ (39)

and

$$\begin{array}{c} |v_{0i}|\leq k_{0i}\left({\left|z_{0i}\right|}^{n/(n+1)}+{\left|y_{i}\right|}^{n/(n+1)}\right)+\left|z_{1i}\right| \end{array}$$ (40)

$$\begin{array}{ccc} & & z_{1i}\left(-k_{1i}{\left|z_{1i}-v_{0i}\right|}^{(n-1)/n}\text{sign}\left(z_{1i}-v_{0i}\right)\right)\\ & & \;\leq k_{1i}\left|z_{1i}\right|{\left|z_{1i}-v_{0i}\right|}^{(n-1)/n}\\ & & \;\leq k_{1i}\left|z_{1i}\right|\left({\left|z_{1i}\right|}^{(n-1)/n}+{\left|v_{0i}\right|}^{(n-1)/n}\right)\\ & & \;\leq k_{1i}{\left|z_{1i}\right|}^{(2n-1)/n}+k_{1i}|z_{1i}|(k_{0i}^{(n-1)/n}\\ & & \;\left({\left|z_{0i}\right|}^{\left(n-1)/(n+1\right)}+{\left|y_{i}\right|}^{\left(n-1)/(n+1\right)}\right)+{\left|z_{1i}\right|}^{(n-1)/n})\\ & & \;=2k_{1i}{\left|z_{1i}\right|}^{(2n-1)/n}+k_{1i}k_{0i}^{(n-1)/n}\\ & & \;(|z_{1i}|{\left|z_{0i}\right|}^{\left(n-1)/(n+1\right)}+|z_{1i}|{\left|y_{i}\right|}^{\left(n-1)/(n+1\right)}) \end{array}$$ (41)

Since the observer Eq (10) can estimate the disturbances within finite time, that is, the estimation errors will converge to zero within finite time, then the estimation errors are bounded, i.e., $|\sigma_{1i}|\leq \sigma_{1i}^{\max}\leq \sigma_{1}^{\max}$. Define

$$\begin{array}{c} \sqrt{y_{1}^{2}+y_{2}^{2}+\cdots +y_{n}^{2}+z_{0}^{T}z_{0}+z_{1}^{T}z_{1}+\cdots +z_{n}^{T}z_{n}}=\eta \end{array}$$ (42)

If *η* > 1, we have |*y*_*i*_| ≤ *η* ≤ *η*^2^, |*z*_*ij*_| ≤ *η* ≤ *η*^2^, |*y*_*i*_*z*_*ij*_| ≤ *η*^2^/2, |*y*_*i*_*y*_*k*_| ≤ *η*^2^/2, |*z*_*ij*_*z*_*kl*_| ≤ *η*^2^/2. Using these inequalities, Eq (37) becomes:

$$\begin{array}{ccc} \dot{M} & \leq & \frac{2(n-1)+1}{2}\eta^{2}+n\eta \sigma_{1}^{\max}+2\gamma_{n}(\eta^{\frac{q_{n}}{q_{n+1}}+1}+\sum_{j=1}^{n-1}\eta^{\frac{q_{n}}{q_{n+1}}+1}+\cdots \\ & & +\gamma_{n-1}^{\frac{q_{n}}{q_{n+1}}}\gamma_{n-2}^{\frac{q_{n-1}}{q_{n+1}}}\cdots \gamma_{1}^{\frac{q_{2}}{q_{n+1}}}\eta^{\frac{1}{q_{n+1}}+1})+n\eta^{2}+\frac{2(n-1)+1+n(n-1)}{2}\eta^{2}\\ & & +\sum_{i=1}^{n}2k_{0i}\eta^{1+n/(n+1)}+\sum_{i=1}^{n}2k_{1i}\eta^{(2n-1)/n}\\ & & +2k_{1i}k_{0i}^{(n-1)/n}\eta^{2n/(n+1)}+\cdots +\sum_{i=1}^{n}k_{ni}\eta \\ & \leq & \frac{n^{2}+5n-2}{2}\eta^{2}+n\eta^{2}\sigma_{1}^{\max}+2\gamma_{n}\left(n+\cdots +\gamma_{n-1}^{\frac{q_{n}}{q_{n+1}}}\gamma_{n-2}^{\frac{q_{n-1}}{q_{n}+1}}\cdots \gamma_{1}^{\frac{q_{2}}{q_{n+1}}}\right)\eta^{2}\\ & & +\sum_{i=1}^{n}2k_{0i}\eta^{2}+\sum_{i=1}^{n}2k_{1i}\eta^{2}+2k_{1i}k_{0i}^{(n-1)/n}\eta^{2}+\cdots +\sum_{i=1}^{n}k_{ni}\eta^{2}=K_{1}M \end{array}$$ (43)

where:

$$\begin{array}{ccc} K_{1} & = & 2(\frac{n^{2}+5n-2}{2}+n\sigma_{1}^{\max}+2\gamma_{n}\left(n+\cdots +\gamma_{n-1}^{\frac{q_{n}}{q_{n+1}}}\gamma_{n-2}^{\frac{q_{n-1}}{q_{n+1}}}\cdots \gamma_{1}^{\frac{q_{2}}{q_{n+1}}}\right)\\ & & +\sum_{i=1}^{n}2k_{0i}+\sum_{i=1}^{n}2k_{1i}+2k_{1i}k_{0i}^{(n-1)/n}+\cdots +\sum_{i=1}^{n}k_{ni}) \end{array}$$ (44)

On the other hand, if *η* ≤ 1, one can find a constant *F*_1_ such that $\dot{M}\leq F_{1}$. Based on above analysis, one can obtain $\dot{M}\leq K_{1}M+F_{1}$. Solving above inequality, one has *M*(*t*) ≤ (*M*(*T*_*u*_) + *F*_1_/*K*_1_)*e*^*K*_1_(*t* − *T*_*u*_)^ − *F*_1_/*K*_1_.

Similarly, we can obtain that for *t* ∈ [0, *T*_*u*_] and *η* > 1, the time derivative of Lyapunov function *M* along Eqs (10) and (12) satisfies:

$$\begin{array}{c} \dot{M}\leq K_{2}M^{\frac{2+\alpha }{2}} \end{array}$$ (45)

While for *η* ≤ 1, one can find a constant *F*_2_ such that $\dot{M}\leq F_{2}$. Solving above inequalities, one has:

$$\begin{array}{c} M\left(t\right)\leq \{\begin{array}{c} {\left(\frac{1}{M{\left(0\right)}^{-\frac{\alpha }{2}}-\frac{\alpha K_{2}}{2}t}\right)}^{\frac{2}{\alpha }}\;\;\;\text{if}\;M\left(0\right)>\frac{1}{2}\\ F_{2}t+M\left(0\right)\;\;\;\;\;\;\text{if}\;M\left(0\right)<\frac{1}{2}\;\text{and}\;M\left(t\right)<\frac{1}{2}\\ {\left(\frac{1}{{\left(\frac{1}{2}\right)}^{-\frac{\alpha }{2}}-\frac{\alpha K_{2}}{2}\left(t-\frac{1/2-M(0)}{F_{2}}\right)}\right)}^{\frac{2}{\alpha }}\;\;\;\text{if}\;M\left(0\right)<\frac{1}{2}\;\text{and}\;M\left(t\right)>\frac{1}{2} \end{array} \end{array}$$ (46)

The states of the System (12) and the observer Eq (10) keep bounded if the switching time satisfies

$$\begin{array}{c} T_{u}<\{\begin{array}{c} \frac{1/2-M(0)}{F_{2}}+\frac{2}{\alpha K_{2}}{\left(\frac{1}{2}\right)}^{-\frac{\alpha }{2}}\;\;\;\text{if}\;M\left(0\right)<\frac{1}{2}\\ \frac{2}{\alpha K_{2}}M{\left(0\right)}^{-\frac{\alpha }{2}}\;\;\;\text{if}\;M\left(0\right)>\frac{1}{2} \end{array} \end{array}$$ (47)

Consequently, for any time interval [0, *t*_1_], the states of the System (12) and the observer Eq (10) will not escape to the infinity.

From above analysis, we can conclude that the composite control scheme consisting of uniformly finite time exact disturbance observer Eq (10) and continuous fixed-time state feedback control law Eq (29) can achieve global stabilization for disturbed nonlinear System (1) within finite time upper bounded by a constant *T*_1_ + *T*_max_ independent of system initial state.

**Remark 7** Theorem 2 shows that the proposed composite control scheme can achieve exact stabilization for disturbed nonlinear systems within finite time upper bounded by a constant independent of initial condition.

**Remark 8** Appropriate value for switching time *T*_*u*_ can be determined through numerical simulation and trial and error. On the one hand, from the proof process of Theorem 1, we need to guarantee the convergence into a compact set *B*_*r*_ = {‖*σ*_*i*_‖ ≤ *r*, *r* > 0} within finite time *T*_*u*_. On the other hand, the selection of switching time *T*_*u*_ needs to ensure the states of the System (12) and the observer Eq (10) keep bounded at any time interval [0, *T*_*u*_].

**Remark 9** In the absence of external disturbances, that is, the disturbances and their all-order derivative are zero, i.e., $d_{i}={\dot{d}}_{i}=\ldots =d_{i}^{\left(n\right)}=0$ (*i* = 1, …, *n*), the observer becomes:

$$\begin{array}{ccc} & & {\dot{\sigma }}_{0i}=-k_{0i}\theta {\left|\sigma_{0i}\right|}^{n/(n+1)}\text{sign}\left(\sigma_{0i}\right)-k_{0i}\left(1-\theta \right){\left|\sigma_{0i}\right|}^{\left(n+1+\alpha )/(n+1\right)}\text{sign}\left(\sigma_{0i}\right)+z_{1i}\\ & & {\dot{z}}_{1i}=-k_{1i}\theta {\left|z_{1i}-{\dot{\sigma }}_{0i}\right|}^{(n-1)/n}\text{sign}\left(z_{1i}-{\dot{\sigma }}_{0i}\right)-k_{1i}\left(1-\theta \right){\left|\sigma_{0i}\right|}^{\left(n+1+2\alpha )/(n+1\right)}\text{sign}\left(\sigma_{0i}\right)+z_{2i}\\ & & ...\\ & & {\dot{z}}_{(n-1)i}=-k_{(n-1)i}\theta {\left|z_{(n-1)i}-{\dot{z}}_{(n-2)i}\right|}^{1/2}\text{sign}\left(z_{(n-1)i}-{\dot{z}}_{(n-2)i}\right)\\ & & \;\;-k_{(n-1)i}\left(1-\theta \right){\left|\sigma_{0i}\right|}^{\left(n+1+n\alpha )/(n+1\right)}\text{sign}\left(\sigma_{0i}\right)+z_{ni}\\ & & {\dot{z}}_{ni}=-k_{ni}\theta \text{sign}\left(z_{ni}-{\dot{z}}_{(n-1)i}\right)-k_{ni}\left(1-\theta \right){\left|\sigma_{0i}\right|}^{1+\alpha }\text{sign}\left(\sigma_{0i}\right) \end{array}$$ (48)

If the initial conditions are selected as *z*_0*i*_(0) = *y*_*i*_(0), *z*_1*i*_(0) = … = *z*_*ni*_(0) = 0, we have *z*_0*i*_(*t*) = *y*_*i*_(*t*) and *z*_1*i*_(*t*) = … = *z*_*ni*_(*t*) = 0 for *t* ≥ 0 and the controller *u* becomes traditional fixed time controller:

$$\begin{array}{c} u=(-\gamma_{n}\left(\xi \right){\left[\xi_{n}\right]}^{1/q_{n+1}}-f\left(y\right))/g\left(y\right) \end{array}$$ (49)

This means that the proposed control scheme acts the same as the baseline fixed time control in the absence of external disturbances, that is, the proposed control scheme retains the nominal performance.

**Remark 10** The proposed control scheme can recover the nominal performance in the absence of disturbances. Further, the proposed control scheme can estimate and compensate the disturbances within uniformly bounded time independent of initial estimation error and achieve fixed-time system stabilization in the presence of disturbances. Therefore, the proposed control scheme improves the disturbance rejection performance.

**Remark 11** Some interesting results have been obtained for nonlinear system control. In [46], a Lyapunov function with adjustable gain coefficient was introduced to control chaotic Josephson junction resonator and force its output to track the target signal. In [47], a modified output feedback neural dynamic surface control was proposed for uncertain MIMO nonlinear system. In [48], an optimal control strategy using adaptive dynamic programming was presented for continuous-time complex-valued nonlinear systems. However, these control schemes cannot achieve exact convergence within finite time. In [49], a *H*_∞_ state feedback control scheme was developed for disturbed and uncertain affine nonlinear discrete-time systems. However, this method considers worst case disturbances, which results in conservative controller design. In [50], a sliding mode controller with system identification observer was presented for position control of medium-stroke voice coil motor. However, under external load disturbance, a tradeoff between disturbance rejection and chattering should be made when selecting sliding mode controller parameters. In [51], a fuzzy *H*_∞_ controller with fuzzy estimator was proposed for a networked control nonlinear system with external disturbances. However, the effect of disturbances on system states can only be attenuated below a desired level. All these control schemes cannot remove the effect of disturbance completely and the performance of nominal system cannot be recovered. In [52], a global finite time observer was designed for uniformly observable and globally Lipschitzian nonlinear systems. However, its estimation time depends on initial condition. The disturbance observer and control scheme proposed in this paper can overcome these problems and achieve uniformly finite time exact disturbance estimation, fixed-time exact system stabilization and performance recovery of nominal system.

## Simulation results

In this section, two illustrative examples are given to demonstrate the effectiveness, the superiority and the applicability of the proposed control scheme.

### Academic example

Consider the following second order system:

$$\begin{array}{c} \left\{ \begin{array}{c} {\dot{x}}_{1}=x_{2}+d_{1}\\ {\dot{x}}_{2}=-\sin\left(x_{1}\right)-2x_{2}+0.25+u+d_{2}\\ y=x_{1} \end{array}\right. \end{array}$$ (50)

**Fig 1:**
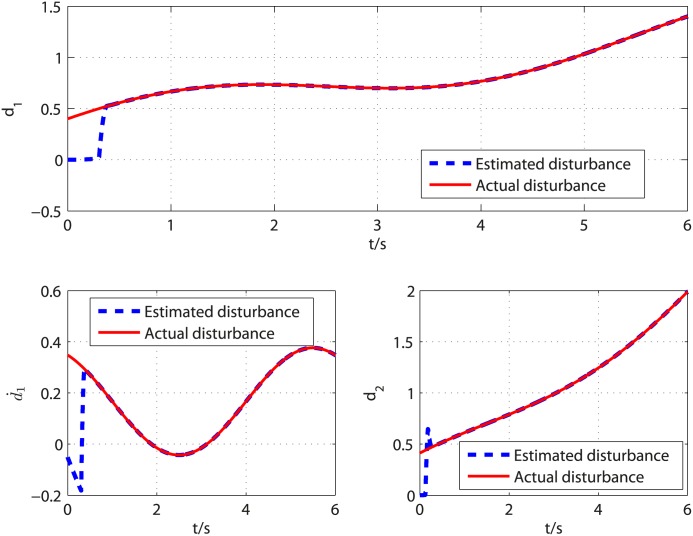
Curves of the disturbances *d*_1_, ${\dot{d}}_{1}$, *d*_2_ and their estimated values under the proposed disturbance observer.

**Fig 2:**
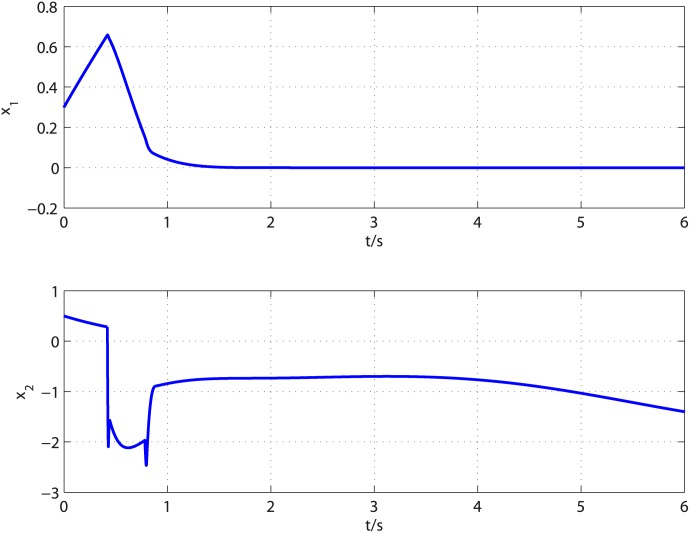
Time response of system states under the proposed control scheme.

**Fig 3:**
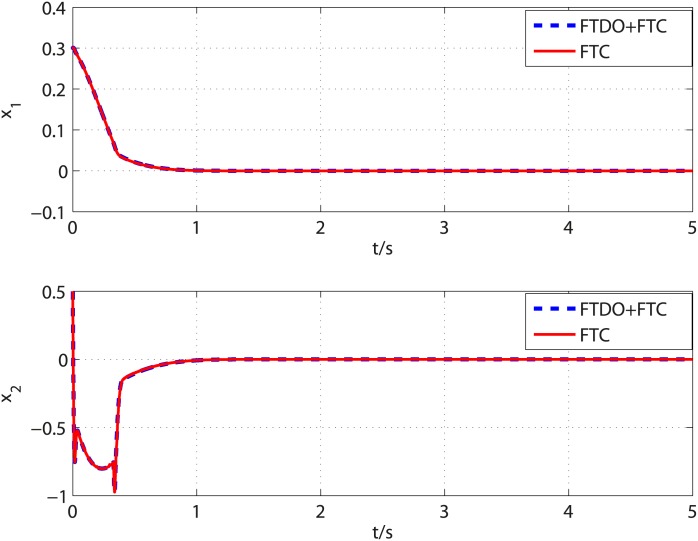
Time response of system states under disturbance observer based control and baseline control.

The disturbances imposed on the System (50) are supposed to be $d_{1}=\frac{t}{6}+0.3+0.2\sin\left(\frac{\pi }{3}t+\frac{\pi }{6}\right)$ and $d_{2}=\frac{t}{3}+0.2+0.3\cos\left(\frac{\pi }{6}t+\frac{\pi }{4}\right)$. The controller parameters are selected to satisfy restricted condition derived in stability analysis and the observer parameters are selected through trial and error. By a careful calculation, the controller parameters are selected as *k*_2_ = 5, *l*_2_ = 3, *k*_1_ = 2, *l*_1_ = 1, *τ* = 17/21. After trial and error, the observer parameters are set to $k_{0i}=8L_{i}^{1/3}$, $k_{1i}=3L_{i}^{2/3}$, *k*_2*i*_ = 1.1*L*_*i*_ (*i* = 1, 2), *L*_1_ = *L*_2_ = 10.5, *T*_*u*_ = 0.303 for *d*_1_ and *T*_*u*_ = 0.126 for *d*_2_. According to [29], the acceptable value for parameter *α* and its upper bound can be determined as follows. In the proof of Theorem 1, one can select a curve *S* = {*σ*_*i*_ ∈ *R*^*n*+1^: *V*(0, *σ*_*i*_) = *δ*, *δ* > 0}, then check whether $\dot{V}\left(\alpha ,\sigma_{i}\right)<0$ holds for that curve with given *α*. For given *α*, if $\dot{V}\left(\alpha ,\sigma_{i}\right)<0$ holds, then the given *α* is called an acceptable value. Starting from *α* = 0 and increasing its value till $\dot{V}\left(\alpha ,\sigma_{i}\right)=0$, the upper bound of parameter *α* can be determined, i.e., the largest value that guarantees $\dot{V}\left(\alpha ,\sigma_{i}\right)<0$. Following the computing method provided in [29], *α* = 0.02 is an acceptable value. The proposed control method is applied to regulate the output of System (50) to the origin. Fig 1 presents the disturbances and their corresponding estimates. It is clear that the observer can give exact disturbances estimation within 0.42 second. The controller is turned on at *t* = 0.42*s* and the response curves of system states are shown in Fig 2. It can be observed that the influence of matched and mismatched disturbances is removed from the output channel and the control objective is accomplished in finite time. Fig 3 displays the response of system states under the proposed uniformly finite time exact disturbance observer based fixed-time control (FTDO + FTC) and baseline fixed-time control (FTC) when there is no disturbance. It can be seen from Fig 3 that the system states under the two controllers are overlapped, which shows that the proposed control method can recover the nominal performance.

**Fig 4:**
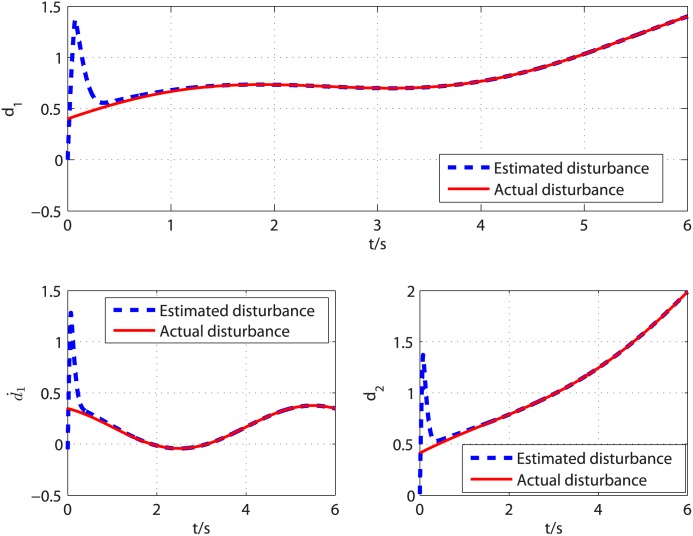
Curves of the disturbances *d*_1_, ${\dot{d}}_{1}$, *d*_2_ and their estimated values under the finite time disturbance observer presented in [26].

**Fig 5:**
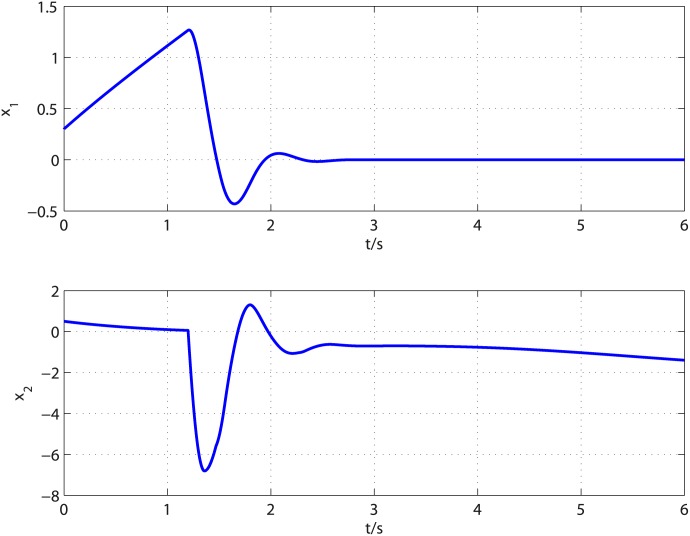
Time response of system states under the control scheme proposed in [26].

**Fig 6:**
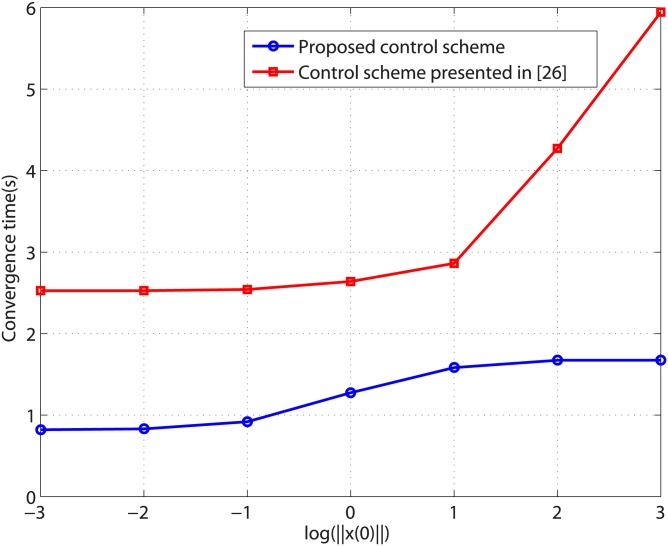
Convergence time versus the logarithm of norm of initial condition.

In order to demonstrate the advantage of the proposed control method, the control scheme proposed in [26] is borrowed to make performance comparison analysis. In [26], finite time disturbance observer is employed to estimate the disturbances. The disturbances and their corresponding estimates are shown in Fig 4. It can be seen from Fig 4 that the observer can give exact disturbances estimation within 1.2 second. The controller is activated at *t* = 1.2*s* and the response curve of system states under the control scheme presented in [26] is shown in Fig 5. As can be seen from Figs 2 and 5, the system response under the proposed control method has less overshoot than that under the control method presented in [26]. Moreover, the settling time of proposed control scheme is shorter than that of the scheme presented in [26]. Fig 6 compares the convergence time of the two controllers under different initial conditions. The results show that the proposed control scheme achieves faster system stabilization. Moreover, the settling time of the control method presented in [26] grows unboundedly with the increment of initial condition, while the convergence time of the proposed control scheme is bounded by a constant as the initial condition increases. Comparative results show that the proposed control scheme has an advantage in convergence time and transient response.

### Application example

**Fig 7:**
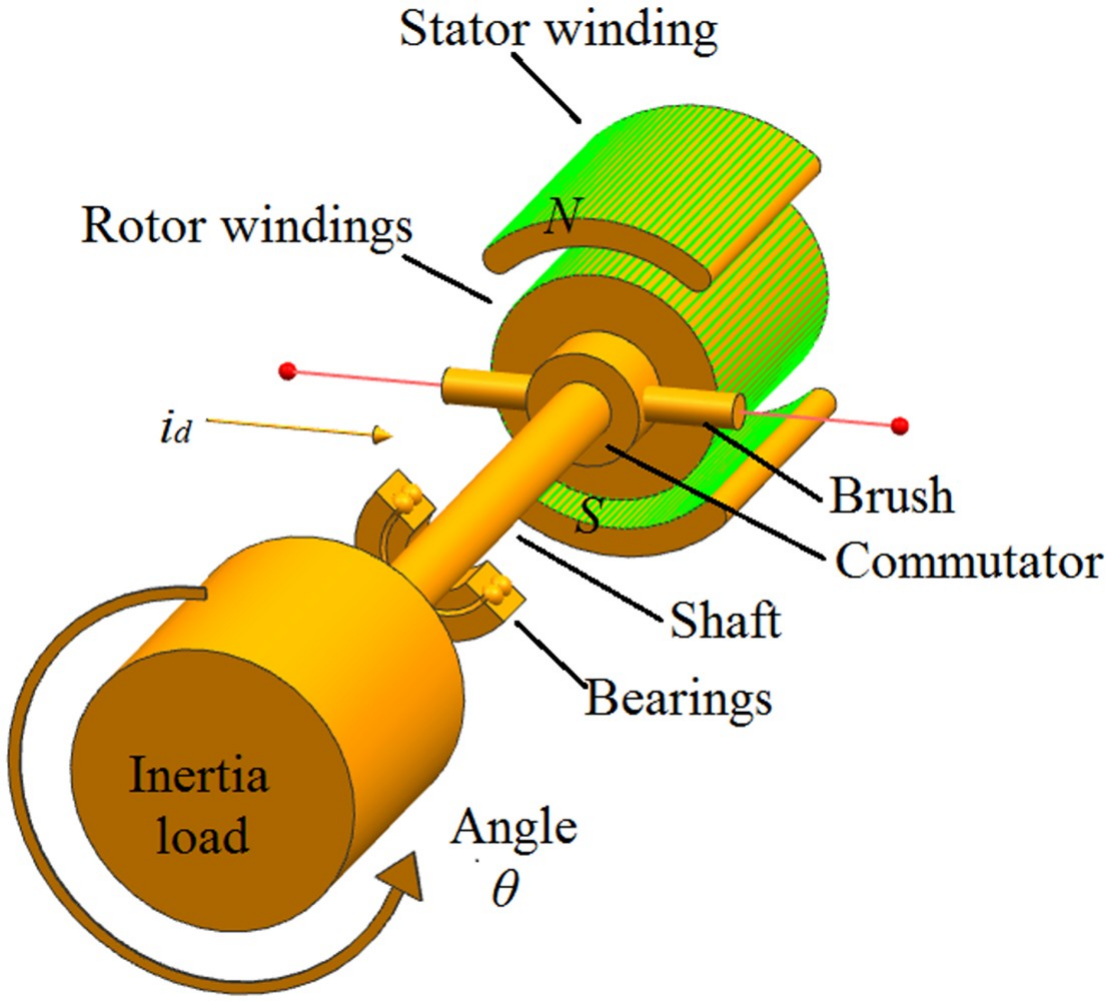
Sketch diagram of DC-motor.

Consider the following classical third order model for the DC-motor shown in Fig 7:

$$\begin{array}{ccc} \dot{\theta }\left(t\right) & = & \omega (t)\\ \dot{\omega }\left(t\right) & = & \frac{1}{J}\left(-b\omega +K_{m}i_{a}\left(t\right)+d_{2}\left(t\right)\right)\\ \frac{di_{a}\left(t\right)}{dt} & = & \frac{1}{L_{a}}\left(-R_{a}i_{a}\left(t\right)-K_{b}\omega \left(t\right)+V_{a}\left(t\right)+d_{3}\left(t\right)\right) \end{array}$$ (51)

where *θ*(*t*) is the rotation angle, *ω*(*t*) denotes the angular velocity, *i*_*a*_(*t*) is the armature current, *V*_*a*_ represents the armature voltage (control input), *J* is the rotor inertia, *K*_*m*_ and *K*_*b*_ are the motor constant and back electromotive force coefficient, *R*_*a*_ and *L*_*a*_ are the armature resistance and the armature inductance, *b* is the friction coefficient, *d*_2_ and *d*_3_ are mismatched and matched disturbances.

Introduce the coordinate transformation *x*_1_ = *θ*, *x*_2_ = *ω*, $x_{3}=\frac{1}{J}\left(-b\omega +K_{m}i_{a}\right)$ and the System (51) becomes:

$$\begin{array}{ccc} {\dot{x}}_{1} & = & x_{2}\\ {\dot{x}}_{2} & = & x_{3}+d_{2}^{{}^{\prime }}\\ {\dot{x}}_{3} & = & -\left(\frac{K_{b}K_{m}}{JL_{a}}+\frac{R_{a}b}{JL_{a}}\right)x_{2}-\left(\frac{b}{J}+\frac{R_{a}}{L_{a}}\right)x_{3}+\frac{K_{m}}{JL_{a}}V_{a}+d_{3}^{{}^{\prime }}\\ x_{o} & = & x_{1} \end{array}$$ (52)

where $d_{2}^{{}^{\prime }}=\frac{d_{2}}{J}$, $d_{3}^{{}^{\prime }}=-\frac{b}{J^{2}}d_{2}+\frac{K_{m}d_{3}}{JL_{a}}$. Now, the system becomes third-order disturbed System (1) with *d*_1_ = 0, $d_{2}=d_{2}^{{}^{\prime }}$, $d_{3}=d_{3}^{{}^{\prime }}$, $f\left(x\right)=-\left(\frac{K_{b}K_{m}}{JL_{a}}+\frac{R_{a}b}{JL_{a}}\right)x_{2}-\left(\frac{b}{J}+\frac{R_{a}}{L_{a}}\right)x_{3}$, $g\left(x\right)=\frac{K_{m}}{JL_{a}}$. We can design continuous control Eq (29) to drive the rotation angle *θ*(*t*) to the origin.

**Fig 8:**
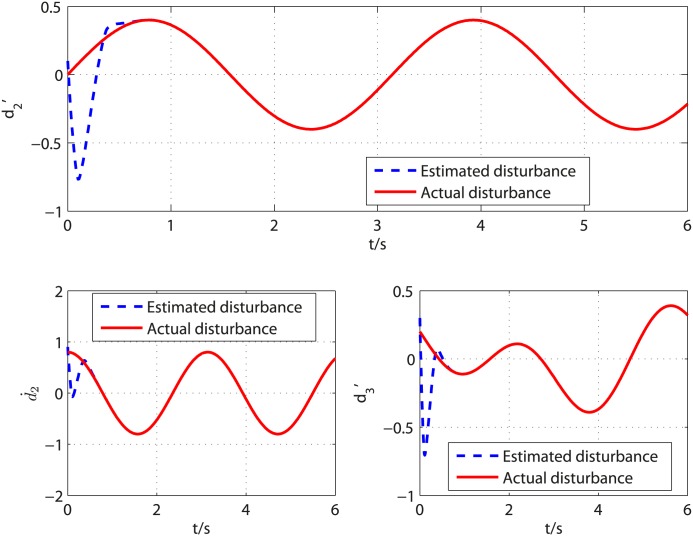
Curves of the disturbances $d_{2}^{{}^{\prime }}$, ${\dot{d}}_{2}^{{}^{\prime }}$, $d_{3}^{{}^{\prime }}$ and their estimated values.

**Fig 9:**
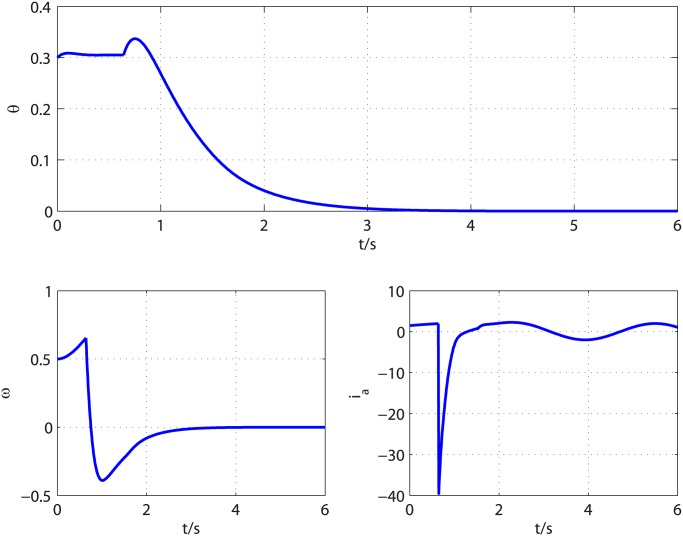
Time response of DC-motor states.

**Fig 10:**
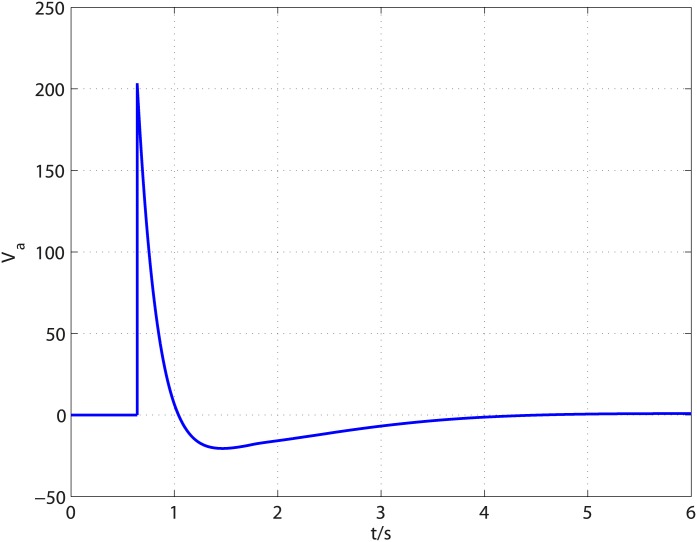
Time response of control input.

The disturbances in System (51) are selected as *d*_2_(*t*) = 0.002*sin*(0.1*t*) and *d*_2_(*t*) = 0.2*cos*(*t*) and the system parameters are chosen as *K*_*b*_ = 0.001, *K*_*m*_ = 0.001, *L*_*a*_ = 0.1, *R*_*a*_ = 0.01, *b* = 0.003, *J* = 0.005. The controller parameters are chosen as *k*_1_ = 1.3, *l*_1_ = 0.1, *k*_2_ = 3, *l*_2_ = 0.4, *k*_3_ = 5, *l*_3_ = 0.8, *τ* = 67/71 and the observer parameters are selected as $k_{0i}=8L_{i}^{1/4}$, $k_{1i}=5L_{i}^{2/4}$, $k_{2i}=3L_{i}^{3/4}$ (*i* = 2, 3), *L*_2_ = *L*_3_ = 3, *T*_*u*_ = 0.14 for *d*_1_ and *T*_*u*_ = 0.26 for *d*_2_. Similar to academic example, *α* = 0.06 is tested to be an acceptable value. Fig 8 shows disturbances $d_{2}^{{}^{\prime }}$, ${\dot{d}}_{2}^{{}^{\prime }}$, $d_{3}^{{}^{\prime }}$ and their corresponding estimates. As shown in Fig 8, the observer can give exact disturbances estimation within 0.64s. The controller is turned on at *t* = 0.64*s* and the results are shown in Fig 9. It is clear that the control objective is accomplished in finite time. The time history of control input *V*_*a*_ is illustrated in Fig 10. It can be observed that the control input is smooth and the amplitude of control input is acceptable for most DC motors.

## Conclusions

The problem of fixed-time stabilization for high order nonlinear systems with matched and mismatched disturbances is investigated via uniformly finite time exact disturbance observer based composite controller design. The composite control strategy presented in this paper is designed based on fixed-time stability theory and adding a power integrator technique, which can regulate the system output to zero within bounded time independent of initial condition. With the aid of constructed Lyapunov function, rigorous global fixed-time stability analysis of closed-loop system is given. Simulation results demonstrate the effectiveness, the superiority and the applicability of the proposed control scheme.

## Appendix

### Appendix A:Proof of Theorem 1

Define estimation error variables: *σ*_0*i*_ = *z*_0*i*_ − *y*_*i*_, $\sigma_{1i}=z_{1i}-d_{i},\ldots ,\sigma_{ni}=z_{ni}-d_{i}^{\left(n-1\right)}$ and the observer error dynamics are governed by:

$$\begin{array}{ccc} & & {\dot{\sigma }}_{0i}=-k_{0i}\theta {\left|\sigma_{0i}\right|}^{n/(n+1)}\mathrm{sign}\left(\sigma_{0i}\right)-k_{0i}\left(1-\theta \right){\left|\sigma_{0i}\right|}^{\left(n+1+\alpha )/(n+1\right)}\mathrm{sign}\left(\sigma_{0i}\right)+\sigma_{1i}\\ & & {\dot{\sigma }}_{1i}=-k_{1i}\theta {\left|\sigma_{1i}-{\dot{\sigma }}_{0i}\right|}^{(n-1)/n}\text{sign}\left(\sigma_{1i}-{\dot{\sigma }}_{0i}\right)-k_{1i}\left(1-\theta \right){\left|\sigma_{0i}\right|}^{\left(n+1+2\alpha )/(n+1\right)}\text{sign}\left(\sigma_{0i}\right)+\sigma_{2i}\\ & & \ldots \\ & & {\dot{\sigma }}_{(n-1)i}=-k_{(n-1)i}\theta {\left|\sigma_{(n-1)i}-{\dot{\sigma }}_{(n-2)i}\right|}^{1/2}\text{sign}\left(\sigma_{(n-1)i}-{\dot{\sigma }}_{(n-2)i}\right)\\ & & \;\;-k_{(n-1)i}\left(1-\theta \right){\left|\sigma_{0i}\right|}^{\left(n+1+n\alpha )/(n+1\right)}\text{sign}\left(\sigma_{0i}\right)+\sigma_{ni}\\ & & {\dot{\sigma }}_{ni}=-k_{ni}\theta \text{sign}\left(\sigma_{ni}-{\dot{\sigma }}_{(n-1)i}\right)-k_{ni}\left(1-\theta \right){\left|\sigma_{0i}\right|}^{1+\alpha }\text{sign}\left(\sigma_{0i}\right)-d_{i}^{\left(n\right)} \end{array}$$ (A.1)

For *t* ≤ *T*_*u*_, the error system becomes:

$$\begin{array}{c} {\dot{\sigma }}_{i}=f_{i}\left(\sigma_{i}\right)+g_{i}\left(\sigma_{i},w\right) \end{array}$$ (A.2)

where *σ*_*i*_ = [*σ*_0*i*_, *σ*_1*i*_, …, *σ*_*ni*_]^*T*^,

$$\begin{array}{cc} f_{i}\left(\sigma_{i}\right)=[\begin{array}{c} -k_{0i}{\left|\sigma_{0i}\right|}^{\left(n+1+\alpha )/(n+1\right)}\mathrm{sign}\left(\sigma_{0i}\right)+\sigma_{1i}\\ -k_{1i}{\left|\sigma_{0i}\right|}^{\left(n+1+2\alpha )/(n+1\right)}\text{sign}\left(\sigma_{0i}\right)+\sigma_{2i}\\ \ldots \\ -k_{(n-1)i}{\left|\sigma_{0i}\right|}^{\left(n+1+n\alpha )/(n+1\right)}\text{sign}\left(\sigma_{0i}\right)+\sigma_{ni}\\ -k_{ni}{\left|\sigma_{0i}\right|}^{1+\alpha }\text{sign}\left(\sigma_{0i}\right) \end{array}] & g_{i}\left(\sigma_{i},w\right)=[\begin{array}{c} 0\\ 0\\ \ldots \\ 0\\ -d_{i}^{\left(n\right)} \end{array}] \end{array}$$

When *g*_*i*_(*σ*_*i*_, *w*) ≡ 0 and *α* = 0, error System (A.1) becomes ${\dot{\sigma }}_{i}=A\sigma_{i}$. Since the matrix *A* is Hurwitz, error state *σ*_*i*_ is asymptotically stable. Select the Lyapunov function *V*(*α*, *σ*_*i*_) = *ξ*_1_(*σ*_*i*_)^*T*^ *Pξ*_1_(*σ*_*i*_) where $\xi_{1}\left(\sigma_{i}\right)={\left[\sigma_{0i}^{1+\alpha },\sigma_{1i}^{\left(n+1+(n+1)\alpha )/(n+1+\alpha \right)},\ldots ,\sigma_{ni}^{\left(n+1+(n+1)\alpha )/(n+1+n\alpha \right)}\right]}^{T}$ and *P* is a symmetric positive definite matrix satisfying *A*^*T*^ *P* + *PA* < 0. Since *V* is proper, *S* = {*σ*_*i*_ ∈ *R*^*n*+1^: *V*(0, *σ*_*i*_) = *δ*, *δ* > 0} is a compact set for arbitrary energy level *δ*. The time derivative of *V*(0, *σ*_*i*_) satisfies:

$$\begin{array}{c} \dot{V}\left(0,\sigma_{i}\right)=\sigma_{i}^{T}\left(A^{T}P+PA\right)\sigma_{i}<0 \end{array}$$ (A.3)

Since $\dot{V}\left(\alpha ,\sigma_{i}\right)$ is continuous in *α* and *σ*_*i*_, $\dot{V}\left(\alpha ,\sigma_{i}\right)$ is uniformly continuous in the set *W* = {(*α*, *σ*_*i*_) ∈ *R* × *R*^*n*+1^|*α* = 0, *σ*_*i*_ ∈ *S*}. This means that there exists a small constant *ε*_1_ such that for all *α* ∈ (0, *ε*_1_), $\dot{V}\left(0,\sigma_{i}\right)<0$ also implies $\dot{V}\left(\alpha ,\sigma_{i}\right)<0$. Therefore, $\dot{V}\left(\alpha ,\sigma_{i}\right)$ is a Lyapunov function of System (A.2) and the error System (A.2) is asymptotically stable. In addition, *f*_*i*_(*σ*_*i*_) is a continuous homogeneous vector field of degree *α* > 0 and the disturbance *g*_*i*_(*σ*_*i*_, *w*) is uniformly bounded by a constant *L*_*i*_. According to Lemma 3, the System (A.2) is practically uniformly convergent, i.e., it can bring arbitrarily large estimation error into a compact set *B*_*r*_ = {*σ*_*i*_: ‖*σ*_*i*_‖ ≤ *r*, *r* > 0} within finite time *T*_*u*_ upper bounded by a constant *t*_*a*_ independent of initial estimation error and the size of this compact set can be prescribed by the designer. After that, the disturbance observer becomes finite time disturbance observer presented in [14]. This means that the observer can give exact disturbance estimation after constant time *t*_*b*_. Therefore, exact disturbance estimation can be achieved within finite time *t*_1_ = *T*_*u*_ + *t*_*b*_ upper bounded by a constant *T*_1_ = *t*_*a*_ + *t*_*b*_ independent of initial estimation error. The proof is completed.

### Appendix B:Proof of proposition 1

It follows from the definition of *W*_*i*+1_ and Lemma 2 that:

$$\begin{array}{ccc} \sum_{j=1}^{i}\frac{\partial W_{i+1}}{\partial {\bar{y}}_{j}}{\dot{\bar{y}}}_{j} & = & -\left(2-\frac{1}{q_{i+1}}\right)\sum_{j=1}^{i}\frac{d{\bar{y}}_{i+1}^{*q_{i+1}}}{d{\bar{y}}_{j}}{\dot{\bar{y}}}_{j}\int_{{\bar{y}}_{i+1}^{*}}^{{\bar{y}}_{i+1}}{\left[s^{q_{i+1}}-{\bar{y}}_{i+1}^{*q_{i+1}}\right]}^{1-1/q_{i+1}}ds\\ & \leq & \left(2-\frac{1}{q_{i+1}}\right){\left|{\bar{y}}_{i+1}^{q_{i+1}}-{\bar{y}}_{i+1}^{*q_{i+1}}\right|}^{1-1/q_{i+1}}|{\bar{y}}_{i+1}-{\bar{y}}_{i+1}^{*}|\sum_{j=1}^{i}|\frac{d{\bar{y}}_{i+1}^{*q_{i+1}}}{d{\bar{y}}_{j}}||{\dot{\bar{y}}}_{j}|\\ & \leq & \left(2-\frac{1}{q_{i+1}}\right){\left|\xi_{i+1}\right|}^{1-1/q_{i+1}}2^{1-1/q_{i+1}}{\left|\xi_{i+1}\right|}^{1/q_{i+1}}\sum_{j=1}^{i}|\frac{d{\bar{y}}_{i+1}^{*q_{i+1}}}{d{\bar{y}}_{j}}||{\dot{\bar{y}}}_{j}| \end{array}$$ (B.1)

Utilizing Eq (20), one has:

$$\begin{array}{ccc} \left|\frac{d{\bar{y}}_{i+1}^{*q_{i+1}}}{d{\bar{y}}_{j}}||{\dot{\bar{y}}}_{j}\right| & = & \left|\frac{d{\bar{y}}_{i+1}^{*q_{i+1}}}{d\xi_{i}}||\frac{d\xi_{i}}{d{\bar{y}}_{i}^{*q_{i}}}||\frac{d{\bar{y}}_{i}^{*q_{i}}}{d\xi_{i-1}}||\frac{d\xi_{i-1}}{d{\bar{y}}_{i-1}^{*q_{i-1}}}\left|\cdots \right|\frac{d{\bar{y}}_{j+1}^{*q_{j+1}}}{d\xi_{j}}||\frac{d\xi_{j}}{d{\bar{y}}_{j}}||{\bar{y}}_{j+1}\right|\\ & \leq & \left||\gamma_{i}{\left(\xi \right)}^{q_{i+1}}\left|+\right|\frac{d\gamma_{i}{\left(\xi \right)}^{q_{i+1}}}{d\xi_{i}}||\xi_{i}\left|||\right|\gamma_{i-1}{\left(\xi \right)}^{q_{i}}\left|+\right|\frac{d\gamma_{i-1}{\left(\xi \right)}^{q_{i}}}{d\xi_{i-1}}||\xi_{i-1}\left||\cdots |\right|\gamma_{j}{\left(\xi \right)}^{q_{j+1}}\right|\\ & & +|\frac{d\gamma_{j}{\left(\xi \right)}^{q_{j+1}}}{d\xi_{j}}||\xi_{j}\left||\right|q_{j}{\bar{y}}_{j}^{q_{j}-1}||{\bar{y}}_{j+1}|=\beta_{ij}\left(\xi \right)|{\bar{y}}_{j}^{q_{j}-1}||{\bar{y}}_{j+1}| \end{array}$$ (B.2)

By Lemma 8 and utilizing Eq (20), one obtains:

$$\begin{array}{c} |{\bar{y}}_{j+1}|\leq {\left|\xi_{j+1}\right|}^{1/q_{j+1}}+\gamma_{j}\left(\xi \right){\left|\xi_{j}\right|}^{1/q_{j+1}} \end{array}$$ (B.3)

$$\begin{array}{c} |{\bar{y}}_{j}^{q_{j}-1}|\leq {\left|\xi_{j}\right|}^{\left(q_{j}-1\right)/q_{j}}+\gamma_{j-1}{\left(\xi \right)}^{q_{j}-1}{\left|\xi_{j-1}\right|}^{\left(q_{j}-1\right)/q_{j}} \end{array}$$ (B.4)

Substituting Eqs (B.3) and (B.4) into Eq (B.2), one has:

$$\begin{array}{ccc} & & \left(2-\frac{1}{q_{i+1}}\right){\left|\xi_{i+1}\right|}^{1-1/q_{i+1}}2^{1-1/q_{i+1}}{\left|\xi_{i+1}\right|}^{1/q_{i+1}}|\frac{d{\bar{y}}_{i+1}^{*q_{i+1}}}{d{\bar{y}}_{j}}||{\dot{\bar{y}}}_{j}|\\ & & \;\leq \left(2-\frac{1}{q_{i+1}}\right)2^{1-1/q_{i+1}}\beta_{ij}\left(\xi \right)\left|\xi_{i+1}\right|\left({\left|\xi_{j+1}\right|}^{1/q_{j+1}}+\gamma_{j}\left(\xi \right){\left|\xi_{j}\right|}^{1/q_{j+1}}\right)\\ & & \;({\left|\xi_{j}\right|}^{\left(q_{j}-1\right)/q_{j}}+\gamma_{j-1}{\left(\xi \right)}^{q_{j}-1}{\left|\xi_{j-1}\right|}^{\left(q_{j}-1\right)/q_{j}})\\ & & \;=\left(2-\frac{1}{q_{i+1}}\right)2^{1-1/q_{i+1}}\beta_{ij}\left(\xi \right)\left|\xi_{i+1}\right|{\left|\xi_{j+1}\right|}^{1/q_{j+1}}{\left|\xi_{j}\right|}^{\left(q_{j}-1\right)/q_{j}}\\ & & \;+\left(2-\frac{1}{q_{i+1}}\right)2^{1-1/q_{i+1}}\beta_{ij}\left(\xi \right)\gamma_{j}\left(\xi \right)\left|\xi_{i+1}\right|{\left|\xi_{j}\right|}^{1/q_{j+1}}{\left|\xi_{j}\right|}^{\left(q_{j}-1\right)/q_{j}}\\ & & \;+\left(2-\frac{1}{q_{i+1}}\right)2^{1-1/q_{i+1}}\beta_{ij}\left(\xi \right)\gamma_{j-1}{\left(\xi \right)}^{q_{j}-1}\left|\xi_{i+1}\right|{\left|\xi_{j+1}\right|}^{1/q_{j+1}}{\left|\xi_{j-1}\right|}^{\left(q_{j}-1\right)/q_{j}}\\ & & \;+\left(2-\frac{1}{q_{i+1}}\right)2^{1-1/q_{i+1}}\beta_{ij}\left(\xi \right)\gamma_{j}\left(\xi \right)\gamma_{j-1}{\left(\xi \right)}^{q_{j}-1}\left|\xi_{i+1}\right|{\left|\xi_{j}\right|}^{1/q_{j+1}}{\left|\xi_{j-1}\right|}^{\left(q_{j}-1\right)/q_{j}} \end{array}$$ (B.5)

Using Lemma 6 and Lemma 7, the first term in Eq (B.5) can be expressed as:

$$\begin{array}{ccc} & & \left(2-\frac{1}{q_{i+1}}\right)2^{1-1/q_{i+1}}\beta_{ij}\left(\xi \right)\left|\xi_{i+1}\right|{\left|\xi_{j+1}\right|}^{1/q_{j+1}}{\left|\xi_{j}\right|}^{\left(q_{j}-1\right)/q_{j}}\\ & & \;\leq \left(2-\frac{1}{q_{i+1}}\right)2^{1-1/q_{i+1}}\beta_{ij}\left(\xi \right)\left|\xi_{i+1}\right|\left(\frac{1/q_{j+1}}{\tau }{\left|\xi_{j+1}\right|}^{\tau }+\frac{\left(q_{j}-1\right)/q_{j}}{\tau }{\left|\xi_{j}\right|}^{\tau }\right)\\ & & \;=\left(2-\frac{1}{q_{i+1}}\right)2^{1-1/q_{i+1}}\beta_{ij}\left(\xi \right)\frac{1/q_{j+1}}{\tau }\left|\xi_{i+1}\right|{\left|\xi_{j+1}\right|}^{\tau }\\ & & \;+\left(2-\frac{1}{q_{i+1}}\right)2^{1-1/q_{i+1}}\beta_{ij}\left(\xi \right)\frac{\left(q_{j}-1\right)/q_{j}}{\tau }\left|\xi_{i+1}\right|{\left|\xi_{j}\right|}^{\tau }\\ & & \;\leq \frac{\tau }{1+\tau }{\left|\xi_{j+1}\right|}^{1+\tau }+\frac{\tau }{1+\tau }{\left|\xi_{j}\right|}^{1+\tau }\\ & & \;+\frac{1}{1+\tau }{\left|\left(2-\frac{1}{q_{i+1}}\right)2^{1-1/q_{i+1}}\beta_{ij}\left(\xi \right)\frac{1/q_{j+1}}{\tau }\right|}^{1+\tau }{\left|\xi_{i+1}\right|}^{1+\tau }\\ & & \;+\frac{1}{1+\tau }{\left|\left(2-\frac{1}{q_{i+1}}\right)2^{1-1/q_{i+1}}\beta_{ij}\left(\xi \right)\frac{\left(q_{j}-1\right)/q_{j}}{\tau }\right|}^{1+\tau }{\left|\xi_{i+1}\right|}^{1+\tau } \end{array}$$ (B.6)

Similarly, we obtain the second, the third and the fourth term in Eq (B.5), whose expressions are Eqs (B.7), (B.8) and (B.9) respectively.

$$\begin{array}{ccc} & & \left(2-\frac{1}{q_{i+1}}\right)2^{1-1/q_{i+1}}\beta_{ij}\left(\xi \right)\gamma_{j}\left(\xi \right)\left|\xi_{i+1}\right|{\left|\xi_{j}\right|}^{1/q_{j+1}}{\left|\xi_{j}\right|}^{\left(q_{j}-1\right)/q_{j}}\\ & & \;\leq \frac{\tau }{1+\tau }{\left|\xi_{j}\right|}^{1+\tau }+\frac{1}{1+\tau }{\left|\left(2-\frac{1}{q_{i+1}}\right)2^{1-1/q_{i+1}}\beta_{ij}\left(\xi \right)\gamma_{j}\left(\xi \right)\right|}^{1+\tau }{\left|\xi_{i+1}\right|}^{1+\tau } \end{array}$$ (B.7)

$$\begin{array}{ccc} & & \left(2-\frac{1}{q_{i+1}}\right)2^{1-1/q_{i+1}}\beta_{ij}\left(\xi \right)\gamma_{j-1}{\left(\xi \right)}^{q_{j}-1}\left|\xi_{i+1}\right|{\left|\xi_{j+1}\right|}^{1/q_{j+1}}{\left|\xi_{j-1}\right|}^{\left(q_{j}-1\right)/q_{j}}\\ & & \;\leq \frac{\tau }{1+\tau }{\left|\xi_{j+1}\right|}^{1+\tau }+\frac{\tau }{1+\tau }{\left|\xi_{j-1}\right|}^{1+\tau }\\ & & \;+\frac{1}{1+\tau }{\left|\left(2-\frac{1}{q_{i+1}}\right)2^{1-1/q_{i+1}}\beta_{ij}\left(\xi \right)\gamma_{j-1}{\left(\xi \right)}^{q_{j}-1}\frac{1/q_{j+1}}{\tau }\right|}^{1+\tau }{\left|\xi_{i+1}\right|}^{1+\tau }\\ & & \;+\frac{1}{1+\tau }{\left|\left(2-\frac{1}{q_{i+1}}\right)2^{1-1/q_{i+1}}\beta_{ij}\left(\xi \right)\gamma_{j-1}{\left(\xi \right)}^{q_{j}-1}\frac{\left(q_{j}-1\right)/q_{j}}{\tau }\right|}^{1+\tau }{\left|\xi_{i+1}\right|}^{1+\tau } \end{array}$$ (B.8)

$$\begin{array}{ccc} & & \left(2-\frac{1}{q_{i+1}}\right)2^{1-1/q_{i+1}}\beta_{ij}\left(\xi \right)\gamma_{j}\left(\xi \right)\gamma_{j-1}{\left(\xi \right)}^{q_{j}-1}\left|\xi_{i+1}\right|{\left|\xi_{j}\right|}^{1/q_{j+1}}{\left|\xi_{j-1}\right|}^{\left(q_{j}-1\right)/q_{j}}\\ & & \;\leq \frac{\tau }{1+\tau }{\left|\xi_{j}\right|}^{1+\tau }+\frac{\tau }{1+\tau }{\left|\xi_{j-1}\right|}^{1+\tau }\\ & & \;+\frac{1}{1+\tau }{\left|\left(2-\frac{1}{q_{i+1}}\right)2^{1-1/q_{i+1}}\beta_{ij}\left(\xi \right)\gamma_{j}\left(\xi \right)\gamma_{j-1}{\left(\xi \right)}^{q_{j}-1}\frac{1/q_{j+1}}{\tau }\right|}^{1+\tau }{\left|\xi_{i+1}\right|}^{1+\tau }\\ & & \;+\frac{1}{1+\tau }{\left|\left(2-\frac{1}{q_{i+1}}\right)2^{1-1/q_{i+1}}\beta_{ij}\left(\xi \right)\gamma_{j}\left(\xi \right)\gamma_{j-1}{\left(\xi \right)}^{q_{j}-1}\frac{\left(q_{j}-1\right)/q_{j}}{\tau }\right|}^{1+\tau }{\left|\xi_{i+1}\right|}^{1+\tau } \end{array}$$ (B.9)

Substituting Eqs (B.6), (B.7), (B.8) and (B.9) into Eq (B.5), we arrive at

$$\begin{array}{ccc} & & \left(2-\frac{1}{q_{i+1}}\right){\left|\xi_{i+1}\right|}^{1-1/q_{i+1}}2^{1-1/q_{i+1}}{\left|\xi_{i+1}\right|}^{1/q_{i+1}}|\frac{d{\bar{y}}_{i+1}^{*q_{i+1}}}{d{\bar{y}}_{j}}||{\dot{\bar{y}}}_{j}|\\ & & \;\leq \frac{2\tau }{1+\tau }{\left|\xi_{j+1}\right|}^{1+\tau }+\frac{3\tau }{1+\tau }{\left|\xi_{j}\right|}^{1+\tau }+\frac{2\tau }{1+\tau }{\left|\xi_{j-1}\right|}^{1+\tau }+\chi_{ij}\left(\xi \right){\left|\xi_{i+1}\right|}^{1+\tau } \end{array}$$ (B.10)

where:

$$\begin{array}{ccc} \chi_{ij}\left(\xi \right) & = & \frac{1}{1+\tau }({\left|\left(2-\frac{1}{q_{i+1}}\right)2^{1-1/q_{i+1}}\beta_{ij}\left(\xi \right)\frac{1/q_{j+1}}{\tau }\right|}^{1+\tau }\\ & & +{\left|\left(2-\frac{1}{q_{i+1}}\right)2^{1-1/q_{i+1}}\beta_{ij}\left(\xi \right)\frac{\left(q_{j}-1\right)/q_{j}}{\tau }\right|}^{1+\tau }\\ & & +{\left|\left(2-\frac{1}{q_{i+1}}\right)2^{1-1/q_{i+1}}\beta_{ij}\left(\xi \right)\gamma_{j}\left(\xi \right)\right|}^{1+\tau }\\ & & +{\left|\left(2-\frac{1}{q_{i+1}}\right)2^{1-1/q_{i+1}}\beta_{ij}\left(\xi \right)\gamma_{j-1}{\left(\xi \right)}^{q_{j}-1}\frac{1/q_{j+1}}{\tau }\right|}^{1+\tau }\\ & & +{\left|\left(2-\frac{1}{q_{i+1}}\right)2^{1-1/q_{i+1}}\beta_{ij}\left(\xi \right)\gamma_{j-1}{\left(\xi \right)}^{q_{j}-1}\frac{\left(q_{j}-1\right)/q_{j}}{\tau }\right|}^{1+\tau }\\ & & +{\left|\left(2-\frac{1}{q_{i+1}}\right)2^{1-1/q_{i+1}}\beta_{ij}\left(\xi \right)\gamma_{j}\left(\xi \right)\gamma_{j-1}{\left(\xi \right)}^{q_{j}-1}\frac{1/q_{j+1}}{\tau }\right|}^{1+\tau }\\ & & +{\left|\left(2-\frac{1}{q_{i+1}}\right)2^{1-1/q_{i+1}}\beta_{ij}\left(\xi \right)\gamma_{j}\left(\xi \right)\gamma_{j-1}{\left(\xi \right)}^{q_{j}-1}\frac{\left(q_{j}-1\right)/q_{j}}{\tau }\right|}^{1+\tau }) \end{array}$$ (B.11)

Substituting Eq (B.10) into Eq (B.1), one has:

$$\begin{array}{ccc} & & \sum_{j=1}^{i}\left(2-\frac{1}{q_{i+1}}\right){\left|\xi_{i+1}\right|}^{1-1/q_{i+1}}2^{1-1/q_{i+1}}{\left|\xi_{i+1}\right|}^{1/q_{i+1}}|\frac{d{\bar{y}}_{i+1}^{*q_{i+1}}}{d{\bar{y}}_{j}}||{\dot{\bar{y}}}_{j}|\\ & & \;\leq \frac{\tau }{1+\tau }{\left|\xi_{2}\right|}^{1+\tau }+\frac{1}{1+\tau }{\left|\left(2-\frac{1}{q_{i+1}}\right)2^{1-1/q_{i+1}}\beta_{1}\left(\xi \right)\right|}^{1+\tau }{\left|\xi_{i+1}\right|}^{1+\tau }\\ & & \;+\frac{\tau }{1+\tau }{\left|\xi_{1}\right|}^{1+\tau }+\frac{1}{1+\tau }{\left|\left(2-\frac{1}{q_{i+1}}\right)2^{1-1/q_{i+1}}\beta_{1}\left(\xi \right)\gamma_{1}\left(\xi \right)\right|}^{1+\tau }{\left|\xi_{i+1}\right|}^{1+\tau }\\ & & \;+\sum_{j=2}^{i-1}\left(\frac{2\tau }{1+\tau }{\left|\xi_{j+1}\right|}^{1+\tau }+\frac{3\tau }{1+\tau }{\left|\xi_{j}\right|}^{1+\tau }+\frac{2\tau }{1+\tau }{\left|\xi_{j-1}\right|}^{1+\tau }+\chi_{ij}\left(\xi \right){\left|\xi_{i+1}\right|}^{1+\tau }\right)\\ & & \;+\frac{\left(q_{i}-1\right)/q_{i}}{1+\tau }{\left|\xi_{i}\right|}^{1+\tau }+\frac{1+1/q_{i+1}}{1+\tau }{\left|\left(2-\frac{1}{q_{i+1}}\right)2^{1-1/q_{i+1}}\beta_{ii}\left(\xi \right)\right|}^{\left(1+\tau \right)/\left(1+1/q_{i+1}\right)}{\left|\xi_{i+1}\right|}^{1+\tau }\\ & & \;+\frac{\left(q_{i}-1\right)/q_{i}}{1+\tau }{\left|\xi_{i-1}\right|}^{1+\tau }+\frac{1+1/q_{i+1}}{1+\tau }|\left(2-\frac{1}{q_{i+1}}\right)2^{1-1/q_{i+1}}\\ & & \;{\beta_{ii}\left(\xi \right)\gamma_{i-1}{\left(\xi \right)}^{q_{i}-1}|}^{\left(1+\tau \right)/\left(1+1/q_{i+1}\right)}{\left|\xi_{i+1}\right|}^{1+\tau }+\frac{\tau }{1+\tau }{\left|\xi_{i}\right|}^{1+\tau }\\ & & \;+\frac{1}{1+\tau }{\left|\left(2-\frac{1}{q_{i+1}}\right)2^{1-1/q_{i+1}}\beta_{ii}\left(\xi \right)\gamma_{i}\left(\xi \right)\right|}^{1+\tau }{\left|\xi_{i+1}\right|}^{1+\tau }+\frac{\tau }{1+\tau }{\left|\xi_{i}\right|}^{1+\tau }\\ & & \;+\frac{\tau }{1+\tau }{\left|\xi_{i-1}\right|}^{1+\tau }+\frac{1}{1+\tau }{\left|\left(2-\frac{1}{q_{i+1}}\right)2^{1-1/q_{i+1}}\beta_{ii}\left(\xi \right)\gamma_{i}\left(\xi \right)\gamma_{i-1}{\left(\xi \right)}^{q_{i}-1}\frac{1/q_{i+1}}{\tau }\right|}^{1+\tau }{\left|\xi_{i+1}\right|}^{1+\tau }\\ & & \;+\frac{1}{1+\tau }{\left|\left(2-\frac{1}{q_{i+1}}\right)2^{1-1/q_{i+1}}\beta_{ii}\left(\xi \right)\gamma_{i}\left(\xi \right)\gamma_{i-1}{\left(\xi \right)}^{q_{i}-1}\frac{\left(q_{i}-1\right)/q_{i}}{\tau }\right|}^{1+\tau }{\left|\xi_{i+1}\right|}^{1+\tau }\\ & & =\sum_{j=1}^{i}c_{(i+1)j}{\left|\xi_{j}\right|}^{1+\tau }+\chi_{i}\left(\xi \right){\left|\xi_{i+1}\right|}^{1+\tau } \end{array}$$ (B.12)

That is,

$$\begin{array}{c} \sum_{j=1}^{i}\frac{\partial W_{i+1}}{\partial {\bar{y}}_{j}}{\dot{\bar{y}}}_{j}\leq \sum_{j=1}^{i}c_{(i+1)j}{\left|\xi_{j}\right|}^{1+\tau }+\chi_{i}\left(\xi \right){\left|\xi_{i+1}\right|}^{1+\tau } \end{array}$$ (B.13)

This completes the proof of proposition 1.
